# Changing the method of consent to increase the numbers of cadaveric donors in Saudi Arabia: the autonomy paradox

**DOI:** 10.12688/f1000research.75994.1

**Published:** 2022-01-17

**Authors:** Deema AL Shawan, Faisal Albagmi, Heba AlNujaidi

**Affiliations:** 1 Public Health, Imam Abdulrahman Bin Faisal University, Dammam, Saudi Arabia; 2 Department of Physical Therapy, Imam Abdulrahman Bin Faisal University, Dammam, Saudi Arabia

**Keywords:** Organ, donation, transplantation, Tawakkalna, consent, cadaveric, donors, family consent

## Abstract

**Background:** In Saudi Arabia, the gap between the demand for and availability of organs persists, with a total of 13,731 patients on the waiting list in 2019. Family refusal is a major obstacle limiting donation since their consent must be obtained prior to the retrieval of organs. The cause of family refusal is mainly due to their lack of knowledge of their loved ones' wish to become a donor. This paper aimed to compare three systems of obtaining consent in terms of effectiveness, respect for autonomy, and the cultural role of families in Saudi Arabia to ensure feasibility and effectiveness in increasing the number of donors.

**Policy alternatives and implications:** The consent systems include informed consent, presumed consent, and mandated choice. The mandated choice policy might be the optimal solution since it is the most likely to balance the respect for individual autonomy and the cultural role of families in Saudi Arabia.

**Conclusions and recommendations:** Mandated choice ensures the respect of autonomy while influencing the next of kin's decision to donate the organs. Additionally, a recommendation to decision makers is to utilize the Tawakkalna app to send alerts to the next of kin when a user registers as a donor with the users' consent. Moreover, more research should be dedicated to investigating the Saudi public's current culture and perceptions towards organ donation to ensure feasibility.

## Introduction

Organ transplantation is one of the major advances of modern medicine: it saves and enhances the quality of the lives of patients with organ failure. Nonetheless, this achievement is hindered by organ shortages worldwide. The gap between the supply and demand of organs is also evident in Saudi Arabia, one of the first Arab countries with an organized organ procurement system ( Shaheen & Souqiyyeh, 2004). Despite Saudi Arabia's efforts to increase the number of donors within the past three decades, the shortage persists, with a total of 13,731 patients who remain on the waiting list in 2019 ( Saudi Center for Organ Transplantation, 2019). The consequences of organ shortage are not limited to the decreased quality and the loss of patients' lives on waiting lists. This public health crisis also has a significant economic impact due to the government funding 48% of dialysis facilities in the Kingdom ( Al-Dossary *et al*., 2013).

The organ procurement system in Saudi Arabia is regulated by The Saudi Center for Organ Transplantation (SCOT), which is a governmental agency. The center was created after the Islamic resolution in 1982, which marked a turning point in the country's history by permitting organ and tissue donation. The main responsibilities of SCOT include allocating organs for transplantation, conducting annual statistics, raising public awareness, and developing policies to ensure the ethical retrieval and transplantation of organs. Additionally, SCOT acts as a referral center for Gulf Cooperation Council (GCC) countries and Spain ( Al-Dossary *et al*., 2013).

There are two main types of donors: 1) living-related and nonrelated; and 2) cadaveric donors. Cadaveric donors are the main source of organs in Saudi Arabia; therefore, the shortage might not be resolved by utilizing living donors ( Alsebayel *et al*., 2004). The current process for organ procurement in Saudi Arabia requires the intensive care unit (ICU) staff, or in some cases, the emergency room (ER) or the surgical unit staff, to identify possible cadaveric donors. A nurse assigned by SCOT notifies the center about the potential deceased donor and documents it. Then, clinical examinations are performed to ensure that the donor meets the brain death criteria developed by SCOT and the Saudi Ministry of Health (MOH). After the process is complete and the declaration of brain death is officially obtained, the ICU physician is required to approach the donor's family to obtain written consent using an official consent form ( Al-Dossary *et al*., 2013).

Possible obstacles that hinder this process include health providers not reporting cases of possible cadaveric donors, family refusal to consent, medical causes, and lack of awareness on the part of hospital staff in donor hospitals. Family refusal is a major obstacle limiting donation, with only 33% of approached families consenting to donate their loved one's organs in 2019 ( Saudi Center for Organ Transplantation, 2019). Lack of knowledge of the potential donors' next of kin of their loved ones' wishes to donate their organs is one of the main reasons for the families' refusal to consent ( Palmer, 2012). To overcome this issue, improvements should be made in documenting potential donors' consent in organ donation registries.

There are three known methods of consent when registering to become a donor upon death: informed consent (opt-in system), mandated choice and presumed consent (opt-out system) ( Al-Dossary *et al*., 2013). This paper aims to analyze the different types of consent systems to address the issue of the high rate of family refusal. The study offers some important insights that will aid decision-makers in selecting an alternative that is more likely to reduce the shortage of organs.

## Policy alternatives and implications

### Informed consent system (status quo)

The current method of registering donors in Saudi Arabia is an opt-in system where individuals voluntarily state that they wish to become donors upon death. Originally, there was no organ donation registry in the Kingdom, and donors were encouraged to fill out donor cards issued by SCOT to document their wishes to be donors upon death. The lack of a registry made it difficult to identify the deceased decision to notify the family unless the donor card was found at the time of death. It is important to note that, under the current policy, families still have the right to veto that decision despite their loved one's consent to become a donor ( Al-Dossary *et al*., 2013).

Donor cards were recently replaced with online registration on SCOT's website as well as the Tawakkalna app. The app was developed by the Saudi Data and Artificial Intelligence Authority (SDAIA) to display the users' coronavirus disease 2019 (COVID-19) status upon entry to public places following government regulations. Due to most of the Saudi public downloading the app, SCOT collaborated with SDAIA to allow users to register to become donors through it ( Yosri, 2021).

In terms of effectiveness, there was a drastic increase of registered donors after the utilization of Tawakkalna. According to a news article, the number of registered donors increased from 20,000 to 200,000, which is a 115% increase after the launch of the donor registration feature. This spike in the number of registered donors was attributed to the app's ease of use and accessibility ( Alsharq Alawsat, 2021).

Nevertheless, when it comes to an opt-in system, there is still a likelihood that willing donors may not register. This issue is prevalent in several countries around the world with a similar opt-in system. For instance, in the United States, 95% of adults support organ donation, while merely 54% are registered as donors ( Anderson, 2017). There are limited studies on this obstacle in Saudi Arabia; nonetheless, one study investigated the willingness of Saudi University students to become donors upon death. According to the results, about 70% of participants are willing to become donors; however, none of them carried donor cards that were the enrollment method for the study ( Al-Ghanim, 2009).

As for ethical considerations, the current opt-in system respects an individual's autonomy. According to Immanuel Kant, undermining a person's autonomy is to treat that person as a mere means to an end without regard for the individual's own goals. Based on Kant's theory, organs from a deceased individual should not be harvested if the person's wish was not to be a donor, even if it intends to save others ( Johnson & Cureton, 2021).

Under informed consent in Saudi Arabia, individuals might still “exercise their autonomy in choosing to accept an institution, tradition, or community that they view as a legitimate source of direction” ( Holm, 2002). Therefore, an organ consent policy needs to ensure that those views are respected.

### Presumed consent system

In a presumed consent system, an individual is assumed to be a donor unless they specifically opt-out. There are two variations of this policy: a hard opt-out and a soft opt-out. In a hard opt-out approach, the families of cadaveric donors are not consulted before retrieving the donated organs. In contrast, in a soft opt-out policy, the families' wishes are considered ( Al-Dossary *et al*., 2013).

Several countries around the world have adopted variations of the presumed consent system with different degrees of success. For instance, after adopting a hard opt-out policy in Austria, the rate of donations quadrupled within eight years ( Zink *et al*., 2005). On the other hand, a soft approach to the policy did not impact donation rates in Spain, which indicates that a hard opt-out policy could be more effective. Despite the success and cost-effectiveness of hard opt-out policies, there are still some concerns, such as increasing the public's mistrust and the risk of undermining autonomy ( Bramhall, 2011).

Applying a hard presumed consent policy in the context of Saudi Arabia might be unfeasible due to ethical considerations pertaining to the role of families. In many cases, the family trumps an individuals' autonomy” ( Al-Shahri, 2002). Therefore, it may be culturally insensitive to implement a hard presumed consent system that disregards the wishes of the next of kin completely, which could risk public acceptance. Additionally, a presumed consent system was found to be the least favored by the Saudi public. One of the causes of this disfavoring was the Islamic belief that a good deed must be done with intent by the individual, which may not be the case in a system that makes you a donor by default. This belief may lead to the public rejecting even a softer presumed consent system as well ( Hammami *et al*., 2012).

### Mandated choice system

Another possible solution is to replace the current policy with a mandated choice system, where citizens are required to either opt-in or opt-out of becoming organ donors. This system can be implemented in Saudi Arabia by utilizing the pre-existing organ donation registration feature on the Tawakkalna app. Currently, the app offers the option to sign up to register as a donor voluntarily; nevertheless, users can unknowingly leave it blank. By implementing a mandated choice system, the app can require individuals to fill out their choice along with the rest of their medical information.

This alternative could increase the number of registered donors by ensuring that willing donors register their wishes and inform their families before death. According to a study conducted in Saudi Arabia in 2018, most participants support organ donation, but merely 2.3% volunteered to carry organ donation cards to indicate their wishes to become donors ( Alnasyan *et al*., 2019). Therefore, this intervention could address the issue of willing donors neglecting to document their decision.

There is sparse evidence on the effectiveness of a mandated choice system in increasing the number of registered donors. However, the limited available research from other countries indicates the likelihood of the success of this system compared to informed and presumed consent. For instance, a study in the Netherlands, a country that adopted a mandated choice, concluded that this system generated more registered donors than both informed and presumed consent systems ( Van Dalen & Henkens, 2014).

As for ethical considerations, a mandated choice system makes the two choices readily available; it increases a person's likelihood of making an autonomous choice and encourages self-determination. In other words, this system eliminates the presumption involved, and each individual could explicitly state their wishes prior to death. Furthermore, this consent mechanism can further cultivate public acceptance due to its respect for families' roles. Additionally, due to the accessibility of the app, individuals can opt-out at any time, which could further ensure the next of kin that this was their loved one's wish with more confidence ( Steffel *et al*., 2019).

Mandated choice is more likely to ensure that families do not go against their loved one's wishes unknowingly by donating their organs. Individuals are less likely to opt-out while they are alive if they have not been given the option of doing so, which may cause their families to possibly give consent after their deaths. Experimental studies suggest that “individuals have more confidence that they know someone else's donation preferences under mandated choice systems than with presumed consent systems” ( Steffel *et al*., 2019, p.77).

## Actionable recommendations

A policy of mandated choice eliminates the presumption involved since an individual can explicitly state their wish to be a donor before death. Therefore, this policy is more likely to be feasible in the Kingdom due to its respect for the cultural role of the family as well.

In addition to changing the method of obtaining consent, there are several other recommendations to maximize the benefits of implementing a mandated choice system.

The Tawakkalna App could be modified to send alerts to notify the next of kin once a user selects their donation decision with the users' permission. Additionally, the users can be asked why they chose to opt-out of donation to investigate the underlying factors influencing donation registration rates.

Decision-makers should invest in more research dedicated to assessing the feasibility of implementing a new consent system. In the case of mandated choice, analyzing the effectiveness of this policy in other countries may not be sufficient evidence. A deep understanding of the current Saudi context, including the public's beliefs, social attitudes, and perceptions, is crucial to predicting the success and cultivating public acceptance for this policy ( Al-Khader *et al*., 2003).

## Conclusion

One of the main obstacles in obtaining family consent in Saudi Arabia is family refusal. The method of obtaining consent could influence the next of kin's decision, especially if it accurately reflects the donor's decision. Due to the cultural role of families in Saudi Arabia, obtaining consent must carefully balance respect for individual autonomy and the role of families. For that reason, mandated choice may be the best alternative to address this moral dilemma.

## Data availability

No data are associated with this article.
