# Changing the method of consent to increase the numbers of cadaveric donors in Saudi Arabia: the autonomy paradox

**DOI:** 10.12688/f1000research.75994.3

**Published:** 2023-06-27

**Authors:** Deema AL Shawan, Faisal Albagmi, Heba AlNujaidi

**Affiliations:** 1 Public Health, Imam Abdulrahman Bin Faisal University, Dammam, Saudi Arabia; 2 Department of Physical Therapy, Imam Abdulrahman Bin Faisal University, Dammam, Saudi Arabia

**Keywords:** Organ, donation, transplantation, Tawakkalna, consent, cadaveric, donors, family consent

## Abstract

**Background:** In Saudi Arabia, the gap between the demand for and availability of organs persists, with a total of 13,731 patients on the waiting list in 2019. Family refusal is a major obstacle limiting donation since their consent must be obtained prior to the retrieval of organs. The cause of family refusal is mainly due to their lack of knowledge of their loved ones' wish to become a donor. This paper aimed to compare three systems of obtaining consent in terms of effectiveness, respect for autonomy, and the cultural role of families in Saudi Arabia to ensure feasibility and effectiveness in increasing the number of donors.

**Policy alternatives and implications:** The consent systems include informed consent, presumed consent, and mandated choice. The mandated choice policy might be the optimal solution since it is the most likely to balance the respect for individual autonomy and the cultural role of families in Saudi Arabia.

**Conclusions and recommendations:** Mandated choice ensures the respect of autonomy while influencing the next of kin's decision to donate the organs. Additionally, a recommendation to decision makers is to utilize the Tawakkalna app to send alerts to the next of kin when a user registers as a donor with the users' consent. Moreover, more research should be dedicated to investigating the Saudi public's current culture and perceptions towards organ donation to ensure feasibility.

## Introduction

Organ transplantation is one of the major advances of modern medicine: it saves and enhances the quality of the lives of patients with organ failure. Nonetheless, this achievement is hindered by organ shortages worldwide. The gap between the supply and demand of organs is also evident in Saudi Arabia, one of the first Arab countries with an organized organ procurement system ( Shaheen & Souqiyyeh, 2004). Despite Saudi Arabia's efforts to increase the number of donors within the past three decades, the shortage persists, with a total of 13,731 patients who remain on the waiting list in 2019 ( Saudi Center for Organ Transplantation, 2019). The consequences of organ shortage are not limited to the decreased quality and the loss of patients' lives on waiting lists. This public health crisis also has a significant economic impact due to the government funding 48% of dialysis facilities in the Kingdom ( Al-Dossary *et al*., 2013).

The organ procurement system in Saudi Arabia is regulated by The Saudi Center for Organ Transplantation (SCOT), which is a governmental agency. The center was created after the Islamic resolution in 1982, which marked a turning point in the country's history by permitting organ and tissue donation. The main responsibilities of SCOT include allocating organs for transplantation, conducting annual statistics, raising public awareness, and developing policies to ensure the ethical retrieval and transplantation of organs. Additionally, SCOT acts as a referral center for Gulf Cooperation Council (GCC) countries and Spain ( Al-Dossary *et al*., 2013).

There are two main types of donors: 1) living-related and nonrelated; and 2) cadaveric donors. Cadaveric donors are the main source of organs in Saudi Arabia; therefore, the shortage might not be resolved by utilizing living donors ( Alsebayel *et al*., 2004). The current process for organ procurement in Saudi Arabia requires the intensive care unit (ICU) staff, or in some cases, the emergency room (ER) or the surgical unit staff, to identify possible cadaveric donors. A nurse assigned by SCOT notifies the center about the potential deceased donor and documents it. Then, clinical examinations are performed to ensure that the donor meets the brain death criteria developed by SCOT and the Saudi Ministry of Health (MOH). After the process is complete and the declaration of brain death is officially obtained, the ICU physician is required to approach the donor's family to obtain written consent using an official consent form ( Al-Dossary *et al*., 2013).

Possible obstacles that hinder this process include health providers not reporting cases of possible cadaveric donors, family refusal to consent, medical causes, and lack of awareness on the part of hospital staff in donor hospitals. Family refusal is a major obstacle limiting donation, with only 33% of approached families consenting to donate their loved one's organs in 2019 ( Saudi Center for Organ Transplantation, 2019). The unknown wish of the donor is one of the reasons for the families' refusal to consent ( Kentish-Barnes *et al.*, 2019; Walker *et al.*, 2013). To overcome this issue, improvements should be made in documenting potential donors' consent in organ donation registries.

There are three known systems of obtaining consent to register donors namely: opt-in, mandated choice, and opt-out systems ( Al-Dossary *et al*., 2013). This paper aims to analyze the different types of consent systems to address the issue of the high rate of family refusal while taking the cultural and ethical context into consideration. The study offers some important insights that will aid decision-makers in selecting an alternative that is more likely to reduce the shortage of organs.

## Policy alternatives and implications

### Opt-in system (status quo)

The current method of registering donors in Saudi Arabia is an opt-in system where individuals voluntarily state that they wish to become donors upon death. Originally, there was no organ donation registry in the Kingdom, and donors were encouraged to fill out donor cards issued by SCOT to document their wishes to be donors upon death. The lack of a registry made it difficult to identify the deceased decision to notify the family unless the donor card was found at the time of death. It is important to note that, under the current policy, families still have the right to veto that decision despite their loved one's consent to become a donor ( Al-Dossary *et al*., 2013).

Donor cards were recently replaced with online registration on SCOT's website as well as the Tawakkalna app. The App was developed by the Saudi Data and Artificial Intelligence Authority (SDAIA) to display the users' coronavirus disease 2019 (COVID-19) status upon entry to public places following government regulations. Due to most of the Saudi public downloading the App, SCOT collaborated with SDAIA to allow users to register to become donors through it ( Tawakkalna, 2020).

After merely 26 days of launching this feature on the App, over 194 thousand users have registered as organ donors. This rapid number of registered donors was attributed to the App's ease of use and accessibility ( *Saudi Press Agency*, 2021).

Nevertheless, when it comes to an opt-in system, there is still a likelihood that willing donors may not register. This issue is prevalent in several countries around the world with a similar opt-in system. For instance, in the United States, 95% of adults support organ donation, while merely 54% are registered as donors ( Anderson, 2017). There are limited studies on this obstacle in Saudi Arabia; nonetheless, one study investigated the willingness of Saudi University students to become donors upon death. According to the results, about 70% of participants are willing to become donors; however, none of them carried donor cards ( Al-Ghanim, 2009).

Some argue that an opt-in system respects personal autonomy since this policy can ensure that the individual's wishes to become a donor is acknowledged ( Farsides, 2012; Taylor, 2005). On the other hand, opponents of opt-in policies state that organs can be obtained from individuals who did not explicitly refuse to donate, but did not express their willingness to do so ( Gill, 2004).

### Presumed consent system

In a presumed consent system, an individual is assumed to be a donor unless they specifically opt-out.

A systematic literature review included studies that investigated the impact on the number of donors before and after implementing this policy in three countries, including Austria, Belgium, and Singapore. The comparative studies have shown that all three countries have increased organ donors ( Rithalia *et al.*, 2009). Nevertheless, it was not effective in other countries, making the resulted inconclusive.

A presumed consent system is ethically sound and may not violate autonomy; nevertheless, there are some concerns. Opponents of this policy argue that it could lead to the removal of organs from individuals who did not want to become donors. On the other hand, the presumption of consent is “morally no worse than not removing organs from the bodies of people who did want them removed” ( Gill, 2004).

However, applying a presumed consent policy in the context of Saudi Arabia might be unfeasible due to ethical considerations pertaining to the role of families. In many cases, the family trumps an individuals' autonomy ( Al-Shahri, 2002). Therefore, it may be culturally insensitive to implement a presumed consent system that disregards the wishes of the next of kin completely, which could risk public acceptance. Additionally, a presumed consent system was found to be the least favored by the Saudi public ( Hammami *et al*., 2012).

### Mandated choice system

Another possible solution is to replace the current policy with a mandated choice system, where citizens are required to either opt-in or opt-out of becoming organ donors. This system can be implemented in Saudi Arabia by utilizing the pre-existing organ donation registration feature on the Tawakkalna app. Currently, the App offers the option to sign up to register as a donor voluntarily; nevertheless, users can unknowingly leave it blank. By implementing a mandated choice system, the App can require individuals to fill out their choice along with the rest of their medical information.

This alternative could increase the number of registered donors by ensuring that willing donors register their wishes and inform their families before death. According to a study conducted in Saudi Arabia in 2018, 77.4% of participants over the age of 40 and 78% of participants under the age of 40 support organ donation. Nevertheless, merely 2.3% volunteered to carry organ donation cards to indicate their wishes to become donors ( Alnasyan *et al*., 2019). Therefore, this intervention could address the issue of willing donors neglecting to document their decision.

There is sparse evidence on the effectiveness of a mandated choice system in increasing the number of registered donors. However, similar policies implemented in other countries can shed some light on its potential effectiveness. For instance, a similar policy was implemented in New South Wales, an Australian state. The policy required individuals to consent or object to become a donor when completing their driver's license application form ( Symons & Poulden, 2022).

The policy had limited success in increasing the number of registered donors and was discontinued in 2012. The discontinuation of that policy was partially due to the public's opposition to this system's potential to increase the number of donors. Nonetheless, such a system could be effective if coupled with an educational campaign ( Symons & Poulden, 2022). Educational campaigns have been proven to increase the number of registered donors ( Deedat *et al.*, 2013). Combining these two interventions could help remedy the issue by minimizing the risk of individuals opting-out due to not being fully knowledgeable about organ donation ( Symons & Poulden, 2022).

Furthermore, individuals are less likely to opt-out while alive if they have not been given the option of doing so, which may cause their families to consent after their deaths. Experimental studies suggest that “individuals have more confidence that they know someone else's donation preferences under mandated choice systems than with presumed consent systems” ( Steffel *et al.*, 2019, p.77). A study conducted in Saudi Arabia ranked mandated choice as the most favored consent system. Nevertheless, the study was limited to a single tertiary hospital, and nation-wide studies must be conducted to further validate this finding ( Hammami *et al.*, 2012).

In terms of ethical concerns, opposing views of mandated choice argue that requiring people to make a choice could undermine autonomy by taking away the right not to choose. Nevertheless, this system makes the two choices readily available; it increases a person's likelihood of making an autonomous choice and encourages self-determination. In other words, this system eliminates the presumption involved, and each individual could explicitly state their wishes before death.

Moreover, religion and culture play an important role in determining how society perceives autonomy ( *Fundamentals of Research on Culture and Psychology: Theory and Methods*, 2016). According to an Islamic belief, a good deed must be done with intent by the individual, which may not be the case in a system that makes you a donor by default ( Hammami *et al.*, 2012). Additionally, due to the accessibility of the App, individuals can opt-out at any time, which could further ensure the next of kin that this was their loved one's wish with more confidence ( Steffel *et al.*, 2019).

## Actionable recommendations

In addition to existing efforts to boost the number of donors, implementing a mandated choice policy could enhance the donor registration feature on the Tawakkalna App. This policy would eliminate the presumption barrier by allowing individuals to explicitly express their desire to be a donor prior to their passing. This approach is more likely to be practical in Saudi Arabia due to the cultural significance that families hold in the Kingdom. Nevertheless, more studies need to be conducted to investigate this policy's feasibility in Saudi Arabia, given its cultural, religious, and social context to ensure its success ( Al-Khader *et al*., 2003).

In addition to changing the method of obtaining consent, there are several other recommendations to maximize the benefits of implementing a mandated choice system. The Tawakkalna App could be modified to send alerts to notify the next of kin once a user selects their donation decision with the users' permission. Additionally, the users can be asked why they chose to opt-out of donation to investigate the underlying factors influencing donation registration rates.

## Conclusion

One of the main obstacles to procuring organs in Saudi Arabia is family refusal. The method of obtaining consent could influence the next of kin's decision, especially if it accurately reflects the donor's decision. Due to the cultural role of families in Saudi Arabia, obtaining consent must carefully balance respect for individual autonomy and the role of families. Therefore, we urge decision-makers to support further research on mandated choice policies as a potentially viable solution to optimize the organ donor registration feature in the Tawakkalna App.

## Data availability

No data are associated with this article.
