# Changing the method of consent to increase the numbers of deceased donors in Saudi Arabia: the autonomy paradox

**DOI:** 10.12688/f1000research.75994.4

**Published:** 2025-03-24

**Authors:** Deema AL Shawan

**Affiliations:** 1 Public Health, Imam Abdulrahman Bin Faisal University, Dammam, Saudi Arabia

**Keywords:** Organ, donation, transplantation, Tawakkalna, consent, cadaveric, donors, family consent

## Abstract

**Background:**

In Saudi Arabia, the gap between the demand for and availability of organs persists, with a total of 13,731 patients on the waiting list in 2019. Family refusal is a major obstacle limiting donation since their consent must be obtained prior to the retrieval of organs. The cause of family refusal is mainly due to their lack of knowledge of their loved ones’ wish to become a donor. This paper aimed to compare three systems of obtaining consent in terms of effectiveness, respect for autonomy, and the cultural role of families in Saudi Arabia to ensure feasibility and effectiveness in increasing the number of donors.

**Policy alternatives and implications:**

The consent systems include informed consent, presumed consent, and mandated choice. The mandated choice policy might be the optimal solution since it is the most likely to balance the respect for individual autonomy and the cultural role of families in Saudi Arabia.

**Conclusions and recommendations:**

Mandated choice could be an option that ensures respect for personal autonomy. Nevertheless, more research should be conducted to support adopting this policy in the Saudi context. Additionally, a recommendation to decision makers is to utilize the Tawakkalna app to send alerts to the next of kin when a user registers as a donor with the user’s consent.

## Introduction

Organ transplantation is one of the major advances of modern medicine: it saves and enhances the quality of the lives of patients with organ failure. Nonetheless, this achievement is hindered by organ shortages worldwide. The gap between the supply and demand of organs is also evident in Saudi Arabia, one of the first Arab countries with an organized organ procurement system ( Shaheen & Souqiyyeh, 2004).

Despite Saudi Arabia’s efforts to increase the number of donors within the past three decades, the shortage persists, with a total of 13,731 patients remaining on the waiting list in 2019 ( Saudi Center for Organ Transplantation, 2019). The consequences of organ shortage are not limited to the decreased quality of life and the loss of patients’ lives on waiting lists. This public health crisis also has a significant economic impact due to the government funding 48% of dialysis facilities in the Kingdom ( Al-Dossary et al., 2013).

The organ procurement system in Saudi Arabia is regulated by The Saudi Center for Organ Transplantation (SCOT), which is a governmental agency. The center was created after the Islamic resolution in 1982, which marked a turning point in the country’s history by permitting organ and tissue donation. The main responsibilities of SCOT include allocating organs for transplantation, conducting annual statistics, raising public awareness, and developing policies to ensure the ethical retrieval and transplantation of organs. Additionally, SCOT acts as a referral center for Gulf Cooperation Council (GCC) countries and Spain ( Al-Dossary et al., 2013). There were various national efforts to increase the number of donors in the Kingdom. These efforts include organ donation awareness campaigns such as the one organized by the Ministry of Health to educate the public on the importance of donation ( Ministry of Health, 2018). Other interventions included a training program to educate healthcare professionals on donor identification and effective communication with potential donors’ families ( The Saudi Center for Organ Transplantation, 2024).

There are two main types of donors: 1) living-related and nonrelated; and 2) deceased donors. Deceased donors are the main source of organs in Saudi Arabia; therefore, the shortage might not be resolved by utilizing living donors ( Alsebayel et al., 2004). The current process for organ procurement in Saudi Arabia requires the intensive care unit (ICU) staff, or in some cases, the emergency room (ER) or the surgical unit staff, to identify possible deceased donors. A nurse assigned by SCOT notifies the center about the potential deceased donor and documents it. Then, clinical examinations are performed to ensure that the donor meets the brain death criteria developed by SCOT and the Saudi Ministry of Health (MOH). After the process is complete and the declaration of brain death is officially obtained, the ICU physician is required to approach the donor’s family to obtain written consent using an official consent form ( Al-Dossary et al., 2013).

Possible obstacles that hinder this process include health providers not reporting cases of possible deceased donors, family refusal to consent, medical causes, and lack of awareness on the part of hospital staff in donor hospitals. In Saudi Arabia, families still hold the right to veto and make the ultimate decision regarding donating their loved one's organs. Therefore, it is crucial to understand what influences their decision. It was found that family refusal is a major obstacle limiting donation, with only 33% of approached families consenting to donate their loved one’s organs in 2019 ( Saudi Center for Organ Transplantation, 2019). The donor’s unknown wish is one of the reasons for the families' refusal to consent ( Kentish-Barnes et al., 2019; Walker et al., 2013). To overcome this issue, improvements should be made in documenting potential donors’ consent in organ donation registries.

In Saudi Arabia, donor cards were recently replaced with online registration on SCOT’s website as well as the Tawakkalna App. The app was developed by the Saudi Data and Artificial Intelligence Authority (SDAIA) to display the users’ coronavirus disease 2019 (COVID-19) status upon entry to public places following government regulations. Due to most of the Saudi public downloading the App, SCOT collaborated with SDAIA to allow users to register to become donors through it (Tawakkalna, 2020). The app is currently used by over 31 million users, as it also provides access to many types of essential services, including healthcare and civil affairs ( Nojood, 2024).

Despite the novelty of the App, after merely 26 days of launching this feature, over 194 thousand users have registered as organ donors. This rapid number of registered donors was attributed to the ease of use and accessibility ( Saudi Press Agency, 2021). Therefore, this App presents a window of opportunity that should be leveraged to ensure that the wishes of willing donors are documented.

For that reason, this paper aims to analyze the different types of consent systems to address the issue of the high rate of family refusal while considering the cultural and ethical context. More specifically, this policy brief compares three systems of obtaining consent to register donors namely: opt-in, mandated choice, and opt-out systems ( Al-Dossary et al., 2013).

## Policy alternatives and implications

### Opt-in system (status quo)

The criteria for analyzing the following alternatives are based on potential effectiveness, as well as cultural and ethical contexts. In particular, the analysis will highlight the unique role of Saudi families in decision-making to address refusals that could stem from the unknown wishes of their loved ones ( Al-Shahri, 2002; Kentish-Barnes et al., 2019; Walker *et al.*, 2013).

The current method of registering donors in Saudi Arabia is an opt-in system where individuals voluntarily state that they wish to become donors upon death. Originally, there was no organ donation registry in the Kingdom, and donors were encouraged to fill out donor cards issued by SCOT to document their wishes to be donors upon death. The lack of a registry made it difficult to identify the deceased decision to notify the family unless the donor card was found at the time of death. It is important to note that, under the current policy, families still have the right to veto that decision ( Al-Dossary et al., 2013).

Nevertheless, when it comes to an opt-in system, there is still a likelihood that willing donors may not register. This issue is prevalent in several countries around the world with a similar opt-in system. For instance, in the United States, 95% of adults support organ donation, while merely 54% are registered as donors ( Anderson, 2017). There are limited studies on this obstacle in Saudi Arabia; nonetheless, one study investigated the willingness of Saudi University students to become donors upon death. According to the results, about 70% of participants were willing to become donors; however, none carried donor cards ( Al-Ghanim, 2009). Another study conducted in Riyadh found that even though participants expressed a strong willingness to donate, only 9.5% of them actually registered as donors, illustrating the disconnect between intention and action ( Almufleh et al., 2018). Even after the launch of the Tawakkalna App, this gap persists. A more recent study found that 21% of respondents registered as donors on the App, despite 87% having a positive view towards being a donor ( Alhasan et al., 2023). These results could exacerbate the rate of family refusal since the donor's unknown wish is one of the main reasons for refusal ( Kentish-Barnes et al., 2019; Walker et al., 2013). Nonetheless, it is important to note that some participants did not register due to their limited awareness of the organ registration policies and the relevant Islamic Fatwas concerning brain death and organ donation. This highlights the potential for increased donation rates if the consent system was accompanied by more educational campaigns aimed at raising awareness in these areas ( Alhasan et al., 2023).

From an ethical prespective, some argue that an opt-in system respects personal autonomy since this policy can ensure that the individual’s wish to become a donor is acknowledged ( Farsides, 2012; Taylor, 2005). Conversely, opponents of opt-in policies argue that an opt-out system could better serve public health by aligning more closely with the presumed wishes of the majority who would support organ donation ( Gill, 2004).

### Presumed consent system

Opt-out systems, in which consent for organ donation is presumed unless individuals explicitly object, have garnered widespread attention due to their potential to increase donation rates. As noted, many studies suggest that countries with opt-out systems tend to perform better in terms of organ donation rates compared to opt-in countries ( Costa-Font et al., 2021; Ugur, 2015). However, most of these studies focus on European contexts, and the cultural nuances and ethical implications of implementing an opt-out system in Saudi Arabia require careful consideration.

A systematic literature review included studies that investigated the impact on the number of donors before and after implementing this policy in three countries, including Austria, Belgium, and Singapore. Comparative studies have shown that all three countries have increased organ donors ( Rithalia et al., 2009).

A presumed consent system may not violate autonomy; nevertheless, there are some concerns. Opponents of this policy argue that it could lead to the removal of organs from individuals who did not want to become donors. On the other hand, the presumption of consent is “morally no worse than not removing organs from the bodies of people who did want them removed” ( Gill, 2004).

Additionally, consent is not necessarily presumed under such legislation but rather provided tacitly. For instance, Saunders (2012) argued that tacit consent is not the same as presumed consent. Rather, it occurs when individuals, aware of the policy and given the opportunity to opt-out, allow their silence or inaction to signify consent. This is particularly relevant in the context of organ donation, where citizens are often made aware of their rights to opt-out, thus allowing for the possibility of informed tacit consent. Therefore, the critique that opt-out systems presume consent without individual input is nuanced by Saunders’ argument that individuals are still able to make a conscious choice, albeit implicitly.

Despite this, it is important to note that while tacit consent may provide an ethically viable justification for opt-out systems in some contexts, cultural considerations—particularly in Saudi Arabia—introduce additional layers of complexity. In Saudi Arabian society, the family trumps an individual’s autonomy ( Al-Shahri, 2002). As such, even under an opt-out system, families may still have significant reservations about consenting to organ donation on behalf of a deceased relative. Therefore, it may be culturally insensitive to implement a presumed consent system that may appear to disregard the wishes of the next of kin, which could risk public acceptance. In fact, a study found that a presumed consent system was found to be the least favored by the Saudi public ( Hammami et al., 2012).

It is also important to note that opt-out systems can vary significantly in how they engage with families ( Pérez, 2023). Some “soft” opt-out models, such as those in Wales, still allow families to play a role in confirming consent or vetoing donation decisions, even when tacit consent has been provided ( Albertsen, 2018). Nevertheless, there are no studies that investigate how the Saudi public views such variations of a presumed consent system. Another critique of opt-out systems as Beraldo and Karpus stated the **“signaling effect”** they produce. By instituting presumed consent, the system can send a societal message about what the “right” thing to do is ( Beraldo & Karpus, 2021). In other words, an opt-out policy implicitly promotes organ donation as the socially desirable norm, placing subtle pressure on individuals to conform to this expectation. Therefore, implementing such a policy may not be a viable option until further research assesses the stakeholder’s awareness and perceptions toward the variation of the presumed consent policy and the potential societal pressure it could create in the Kingdom.

### Mandated choice system

Another possible solution is to replace the current policy with a mandated choice system, where citizens are required to either opt-in or opt-out of becoming organ donors. This system can be implemented in Saudi Arabia by utilizing the pre-existing organ donation registration feature on the Tawakkalna App. Currently, the App offers the option to sign up to register as a donor voluntarily; nevertheless, users can unknowingly leave it blank. By implementing a mandated choice system, the App can require individuals to fill out their choice along with the rest of their medical information.

According to a study conducted in Saudi Arabia in 2018 found that 77.4% of participants over the age of 40 and 78% of those under 40 support organ donations. However, only 2.3% of participants volunteered to carry organ donation cards to indicate their intentions to become donors ( Alnasyan et al., 2019). Furthermore, more recent research conducted after the launch of the App found that 22.2% of participants did not register as donors because they were unaware of that option being available ( Alhasan et al., 2023). Therefore, this intervention could aid in decreasing the number of willing donors not documenting their decisions.

Furthermore, studies conducted in other countries can shed some light on potential effectiveness. For instance, a similar policy was implemented in New South Wales, an Australian state. The policy required individuals to consent or object to becoming a donor when completing their driver’s license application form. The policy had limited success in increasing the number of registered donors and was discontinued in 2012. However, this decision was partly influenced by public concerns that the system might hinder registration. Many individuals opted for “no” due to their unfamiliarity with the policy. Therefore, such a system could be more effective if accompanied by an educational campaign to address and overcome these obstacles ( Symons & Poulden, 2022). Educational campaigns have been proven to increase the number of registered donors ( Deedat et al., 2013). Combining these two interventions could help remedy the issue by minimizing the risk of individuals opting-out due to not being fully knowledgeable about organ donation ( Symons & Poulden, 2022).

Although educational campaigns can potentially enhance informed consent, ethical, religious, and cultural concerns may make them less favorable to the public. For instance, individuals are less likely to opt out while alive if they have not been allowed to do so, which may lead their families to consent after their deaths. Experimental studies indicate that “individuals have more confidence that they know someone else’s donation preferences under mandated choice systems than with presumed consent systems” ( Steffel et al., 2019, p. 77). Additionally, a study conducted in Saudi Arabia found that mandated choice was the most favored consent system ( Hammami et al., 2012).

As for religious influences, Islamic belief holds that a good deed must be performed with conscious intent. Consequently, implementing a mandated choice model respects this religious perspective by ensuring that individuals make a conscious decision about organ donation. It is also essential that this consent system is accompanied by an educational campaign that clarifies Fatwas and religious rulings related to organ donation. Additionally, the accessibility of the App allows individuals to opt-out at any time, which could further reassure next of kin that this was truly their loved one’s wish ( Steffel et al., 2019).

## Actionable recommendations

Implementing a mandated choice policy could enhance the donor registration feature on the Tawakkalna App. This policy would eliminate the presumption barrier by allowing individuals to explicitly express their desire to be a donor. This approach is more likely to be practical in Saudi Arabia due to the cultural significance that families hold in the Kingdom.

In addition to changing the method of obtaining consent, several recommendations can maximize the benefits of the Tawakkalna App. One key improvement is to introduce an option for organ donation that allows users to delegate the decision to their chosen next of kin. This additional feature could help address concerns about respecting individuals’ rights not to decide. Furthermore, the app could serve as an effective tool for collecting data on why users opt out of donation, enabling an investigation into the underlying factors that influence registration rates. Lastly, the new consent system should be accompanied by an educational campaign aimed at correcting common misconceptions about organ donation, thereby enhancing the effectiveness of this policy ( Alhasan et al., 2023).

Furthermore, future research should investigate all factors that can impact the number of organ donors. For instance, more studies should aim to evaluate the current process of organ referrals to the Saudi Center for Organ Transplantation (SCOT) to ensure that all patient deaths are reported, thereby increasing the number of potential donors and the rate of family consent. Upcoming studies should also focus on assessing the effectiveness of training healthcare providers in discussing the donation of their loved ones’ organs, as this is another important element in improving consent rates ( Timar et al., 2021). Lastly, more studies need to be conducted to investigate this policy’s feasibility in Saudi Arabia, given its cultural, religious, and social context to ensure its success ( Al-Khader et al., 2003).

## Conclusion

One of the main obstacles to procuring organs in Saudi Arabia is family refusal. The method of obtaining consent could influence the next of kin’s decision, especially if it accurately reflects the donor’s decision. Due to the cultural role of families in Saudi Arabia, obtaining consent must carefully balance respect for individual autonomy and the role of families. Therefore, we urge decision-makers to support further research on mandated choice policies as a potentially viable solution to optimize the organ donor registration feature in the Tawakkalna App.

## Data availability

No data are associated with this article.
