# Supplementary material for: Changing the method of consent to increase the numbers of deceased donors in Saudi Arabia: the autonomy paradox
Source: F1000Res. 2025 Mar 24;11:53. Originally published 2022 Jan 17. [Version 4] doi: 10.12688/f1000research.75994.4 (PMC12365590; doi:10.12688/f1000research.75994.4)
Supplement: Peer review history

# Reviewer report: Reviewer response for version 4

**DOI:** 10.5256/f1000research.175759.r396834

**Published:** 2025-08-19

**Authors:** Md Shaikh Farid

**Affiliations:** 1 Dhaka University, Dhaka, Bangladesh

The manuscript titled *"Changing the method of consent to increase the numbers of deceased donors in Saudi Arabia: the autonomy paradox"* addresses a timely and ethically complex question. It explores whether shifting from an opt-in to an opt-out consent system for deceased organ donation in Saudi Arabia would not only increase donation rates but paradoxically promote personal autonomy within an Islamic context. This is a compelling and original argument. However, the central thesis—namely, that presumed consent may enhance rather than diminish autonomy in this particular sociocultural and religious setting—needs to be more clearly articulated and developed. The author introduces the concept of an “autonomy paradox” but does not adequately define or trace how this concept aligns with both Western and Islamic philosophical frameworks.

 The paper references some comparative international practices and Islamic legal principles but lacks a detailed engagement with context-specific evidence. The discussion would benefit from more empirical data on Saudi public opinion, fatwas from relevant Islamic authorities, and current legal frameworks surrounding organ donation in the country. Without this, the normative claims risk being too speculative. The Islamic bioethical reasoning would also be stronger if the author demonstrated a deeper engagement with classical jurisprudential sources (fiqh) and contemporary religious discourse in Saudi Arabia.

 Structurally, the manuscript has promise but suffers from weak transitions between philosophical argument and policy implications. The introduction needs a more focused research question, and the conclusion would be more effective if it clearly restated the argument and reflected on its broader ethical and policy consequences. Currently, the sections read somewhat independently of one another rather than as parts of a coherent argumentative arc.

 Key concepts such as “autonomy,” “Islamic ethics,” and “presumed consent” should be clearly defined and used consistently throughout. Greater attention to nuance in how autonomy is conceptualized in Islamic versus liberal frameworks would sharpen the philosophical dimension of the paper.

 In sum, this is a promising and original manuscript that engages with an underexplored ethical issue in Islamic bioethics and organ donation policy. However, substantial revisions are needed to clarify the argument, strengthen the evidence base, and improve coherence and terminology. I recommend a major revision before the manuscript can be considered for indexing.

Does the paper provide a comprehensive overview of the policy and the context of its implementation in a way which is accessible to a general reader?

Partly

Is the discussion on the implications clearly and accurately presented and does it cite the current literature?

Partly

Are the recommendations made clear, balanced, and justified on the basis of the presented arguments?

Partly

Reviewer Expertise:

The manuscript titled "Changing the method of consent to increase the numbers of deceased donors in Saudi Arabia: the autonomy paradox" addresses a timely and ethically complex question. It explores whether shifting from an opt-in to an opt-out consent system for deceased organ donation in Saudi Arabia would not only increase donation rates but paradoxically promote personal autonomy within an Islamic context. This is a compelling and original argument. However, the central thesis—namely, that presumed consent may enhance rather than diminish autonomy in this particular sociocultural and religious setting—needs to be more clearly articulated and developed.The author introduces the concept of an “autonomy paradox” but does not adequately define or trace how this concept aligns with both Western and Islamic philosophical frameworks.The paper references some comparative international practices and Islamic legal principles but lacks a detailed engagement with context-specific evidence. The discussion would benefit from more empirical data on Saudi public opinion, fatwas from relevant Islamic authorities, and current legal frameworks surrounding organ donation in the country. Without this, the normative claims risk being too speculative. The Islamic bioethical reasoning would also be stronger if the author demonstrated a deeper engagement with classical jurisprudential sources (fiqh) and contemporary religious discourse in Saudi Arabia.Structurally, the manuscript has promise but suffers from weak transitions between philosophical argument and policy implications. The introduction needs a more focused research question, and the conclusion would be more effective if it clearly restated the argument and reflected on its broader ethical and policy consequences. Currently, the sections read somewhat independently of one another rather than as parts of a coherent argumentative arc.Key concepts such as “autonomy,” “Islamic ethics,” and “presumed consent” should be clearly defined and used consistently throughout. Greater attention to nuance in how autonomy is conceptualized in Islamic versus liberal frameworks would sharpen the philosophical dimension of the paper.In sum, this is a promising and original manuscript that engages with an underexplored ethical issue in Islamic bioethics and organ donation policy. However, substantial revisions are needed to clarify the argument, strengthen the evidence base, and improve coherence and terminology. I recommend a major revision before the manuscript can be considered for publication.

I confirm that I have read this submission and believe that I have an appropriate level of expertise to confirm that it is of an acceptable scientific standard, however I have significant reservations, as outlined above.

# Reviewer report: Reviewer response for version 4

**DOI:** 10.5256/f1000research.175759.r396843

**Published:** 2025-08-11

**Authors:** Noble Gracious

**Affiliations:** 1 Govt Medical College, Alappuzha, Kerala, India

Approved because of its relevance. It is a well conceived study addressing the demand supply gap for organs to the patients with end stage organ failure. Relooking the consent system to ensure the subjects autonomy is addressed in the study. The assent of the relatives at the time of donation is advisable, but the author has not addressed the matter in the study

Does the paper provide a comprehensive overview of the policy and the context of its implementation in a way which is accessible to a general reader?

Yes

Is the discussion on the implications clearly and accurately presented and does it cite the current literature?

Yes

Are the recommendations made clear, balanced, and justified on the basis of the presented arguments?

Yes

Reviewer Expertise:

Policy research in end stage renal failure,deceased organ transplantation,transplant immunology,diabetes and kidney disease

I confirm that I have read this submission and believe that I have an appropriate level of expertise to confirm that it is of an acceptable scientific standard.

# Reviewer report: Reviewer response for version 4

**DOI:** 10.5256/f1000research.175759.r372677

**Published:** 2025-04-08

**Authors:** Andreas Albertsen

**Affiliations:** 1 Aarhus University, Aarhus, Denmark

I appreciate the author's efforts in clarifying their proposal and in accommodating the received comments. To me at least, the proposal is sufficiently clear at this point. A few minor suggestions:

 - Regarding the family veto. This term is typically reserved for cases where the deceased has registered as a donor; the family refuses and the latter is decisive.But it is, of course, still true that family refusals are an important source for people's organs not being utilized.

 - Author write 'As such, even under an opt-out system, families may still have significant reservations about consenting to organ donation on behalf of a deceased relative. Therefore, it may be culturally insensitive to implement a presumed consent system that may appear to disregard the wishes of the next of kin, which could risk public acceptance. '

 This seems to be two different problems written as one. It might be culturally insensitive to implement an opt-out system that disregards next of kind, but then if we try to accomodate this through a soft opt-out system, we risk that next of kin will not consent to donation (But that is not because they are disregarded, if they were they would not be given the choice.

 Regarding mandated choice. Might be worth highlighting the impressive share of the population that uses the app. Right now you just mention the absolute number, but some readers will not know how larges a proportion of the citizens that is.

 One important specification that I found lacking is in what sense the choice is going to be mandated. Traditionally, mandated choice policies meant that you HAD to choose. If you did not choose you would loose something of value (i.e. access to a drivers licence).

 Is this mandated choice in the same sense? What is lost here. The ability to use the app? If so, how big a deal that is depends on how important it is. Is it the only way to access something important? Will not having the app be inconvenient, or something worse?

 If not much is at stake, then the proposal becomes more of what some call  an active choice policy (or incomplete mandated choice policy), where people are asked to choose (but not forced to do so).

Does the paper provide a comprehensive overview of the policy and the context of its implementation in a way which is accessible to a general reader?

Partly

Is the discussion on the implications clearly and accurately presented and does it cite the current literature?

Partly

Are the recommendations made clear, balanced, and justified on the basis of the presented arguments?

Yes

Reviewer Expertise:

Ethics of organ donation; political science, political philosophy

I confirm that I have read this submission and believe that I have an appropriate level of expertise to confirm that it is of an acceptable scientific standard, however I have significant reservations, as outlined above.

# Reviewer report: Reviewer response for version 3

**DOI:** 10.5256/f1000research.149488.r202382

**Published:** 2023-10-26

**Authors:** Cynthia Isenor

**Affiliations:** 1 Nova Scotia Health Authority, Nova Scotia, Canada

This article has foundational elements to provide a comprehensive discussion on the need for change in the method for registered consent decisions for deceased donation in Saudi Arabia. Some suggestions below for enhancement may be limited due to word counts, consideration on alternative language may be required by the authors. 

 Overall, there are statements about the donation and transplantation system in Saudi Arabia, but very little specifics on what this system looks like; see introduction paragraph 1, sentence 4 as an example of this. Timar et al. (2021) and Williment et al. (2023) provide comprehensive overviews of key elements for successful donation programs: optimizing referral is a key element to increasing donation rates, as more donors are referred, more families are offered the opportunity (increased approach) and therefore more consent occurs. Having individuals who are specially trained in discussion donation is another key element for improvement. Registered consent decisions is yet another element. 

**Introduction**

 Clarity on the 'efforts to improve Saudi Arabia's donor rates' is required in this section as per the above. 

 The connection between the need for policy change on registered consent decisions and the impact to improving donation in Saudi Arabia requires more data on the overall donation system in Saudi Arabia; this would be one key method for improving the rate of donation, without this data it is challenging to determine this is 'a major obstacle limiting donation'.

**Policy alternatives and implications**

 There is some brief, pertinent, and interesting information stated about the role of family in the Saudi Arabian culture. More emphasis on the role of the family in end of life care decisions, including donation, should be considered as preface to the differing policy alternatives. This will enable the reader to truly understand the recommendations, why they are sound from a cultural-family perspective, and how the authors envision this to increase overall rates.

**Opt-in**

 The authors describe the current system which is a soft consent opt-in system; one in which a registered decision can be overturned by a family (Etheredge 2022). Updated statistics on the overall rate of registry would be recommended; the limited data on the success of ‘the App’ is impressive but stands on only 6 days of data and a very limited portion of the Saudi Arabian population. The overall argument against the status quo is not evident; exploration on the use/amount of public education would help justify the deviation from the status quo, for example.

**Presumed consent system**

 Paragraph 2 requires literature to support/strengthen the last sentence on countries where this was not effective.

 The last paragraph in this section becomes compelling on why this approach will not work in Saudi Arabia. To strengthen this point, I would suggest adding some literature on the impact to losing public trust if this method were to become policy. Additionally, data to support the last sentence is recommended.

**Mandated choice system**

 Capitalizing the Tawakkalna app requires data to demonstrate that the App is widely accessible for the large majority of the population.

 Paragraph 4 is the beginning of a compelling argument for enhanced public awareness in combination with a consent system, not just mandated choice. The same could be stated for the status quo.

 This section lacks cohesive flow. Paragraph 2 is incomplete; why does this approach ensure registration and information sharing with families? The last paragraph, sentence 2, begins to demonstrate why in Saudi Arabian culture, the use of mandated choice may be supported, “According to an Islamic belief, a good deed must be done with intent by the individual…”. Use this concept and literature that demonstrates how this improves autonomy of decisions to support the mandated choice approach, if this the best approach to be used.

 Overall: This work is extremely important to increase the donation and transplantation communities understanding of donation decisions for people and families from Saudi Arabia and to help determine the next steps for policy reform.   

 References

 Timar, J., Bleil, M., Daly, T., Koomar, S., Hasz, R., & Nathan, H. (2021). Successful strategies to increase organ donation: the Gift of Life Donor Program Philadelphia model. Indian Journal of Thoracic and Cardiovascular Surgery, 37 (Suppl 3): S380-394. .

https://doi.org/10.1007/s12055-021-01219-9.

 Williment, C., Beaulieu, L., Clarkson, A., Gunderson, S., Hartell, D., Escoto, M. et al. (2023). Organ Donation Organization Architecture: Recommendations From an International Consensus Forum. Transplantation Direct 9(5):e1440.

 DOI: 10.1097/TXD.0000000000001440.

 Etheredge, H.R. (2021). Assessing Global Organ Donation Policies: Opt-In vs Opt-Out. Risk Management and Healthcare Policy, 14; 1985-1998. https://doi.org/10.2147/RMHP.S270234.

Does the paper provide a comprehensive overview of the policy and the context of its implementation in a way which is accessible to a general reader?

Partly

Is the discussion on the implications clearly and accurately presented and does it cite the current literature?

No

Are the recommendations made clear, balanced, and justified on the basis of the presented arguments?

No

Reviewer Expertise:

Deceased donation reform; deceased donation best practices

I confirm that I have read this submission and believe that I have an appropriate level of expertise to state that I do not consider it to be of an acceptable scientific standard, for reasons outlined above.

## References

[1] : Successful strategies to increase organ donation: the Gift of Life Donor Program Philadelphia model. *Indian J Thorac Cardiovasc Surg* .2021;37(Suppl 3) : 10.1007/s12055-021-01219-9 380-394 10.1007/s12055-021-01219-9 34629767 PMC8464643

[2] : Organ Donation Organization Architecture: Recommendations From an International Consensus Forum. *Transplant Direct* .2023;9(5) : 10.1097/TXD.0000000000001440 e1440 10.1097/TXD.0000000000001440 37138552 PMC10150918

[3] : Assessing Global Organ Donation Policies: Opt-In vs Opt-Out. *Risk Manag Healthc Policy* .2021;14: 10.2147/RMHP.S270234 1985-1998 10.2147/RMHP.S270234 34012308 PMC8128443

# Response

**Published:** 2025-02-27

**Authors:** Deema AL Shawan

**Affiliations:** Imam Abdulrahman Bin faisal University, Saudi Arabia

Dear reviewer,

 Thank you for your valuable feedback.

  I have incorporated your suggested edits into the revised manuscript. Additionally, you will find our detailed responses to each of your comments below.

**Comment:**

**“Overall, there are statements about the donation and transplantation system in Saudi Arabia, but very little specifics on what this system looks like; see introduction paragraph 1, sentence 4 as an example of this. Timar et al. (2021) and Williment et al. (2023) provide comprehensive overviews of key elements for successful donation programs: optimizing referral is a key element to increasing donation rates, as more donors are referred, more families are offered the opportunity (increased approach) and therefore more consent occurs. Having individuals who are specially trained in discussion donation is another key element for improvement. Registered consent decisions is yet another element. “**

**Response: **Thank you for your insightful feedback.

 We agree that optimizing referral systems and training healthcare professionals on how to discuss donations with potential donors' families significantly impact increasing donation rates. 

 The policy brief does not attempt to provide an overview of all interventions implemented to boost donation rates in Saudi Arabia. Instead, our focus was on leveraging the opportunity presented by the Tawakkalna App, which enables millions of users to register their donation wishes. We aimed to ensure this initiative is as impactful as possible in addressing one of the main obstacles to donation: family refusal due to the lack of knowledge of their loved one’s wish.  However, to provide contextual information we took your suggestion and added other efforts by the Saudi government to enhance donation rates.

 We also concur that all elements that could enhance donation rates should be considered, which is why we incorporated your suggestions as important  implication for further research under the actionable recommendation section.

**Comment:**

**“Introduction**

**Clarity on the 'efforts to improve Saudi Arabia's donor rates' is required in this section as per the above.**

**The connection between the need for policy change on registered consent decisions and the impact to improving donation in Saudi Arabia requires more data on the overall donation system in Saudi Arabia; this would be one key method for improving the rate of donation, without this data it is challenging to determine this is 'a major obstacle limiting donation'.”** 

**Response: **Thank you and the efforts of other efforts were added to the introduction and suggestion were made for further research under the recommendation section due to the sparse evidence in Saudi Arabia.

 In terms of the data requested, we added that information under the Informed Consent section (status quo).

**Comment**

**“Policy alternatives and implications**

**There is some brief, pertinent, and interesting information stated about the role of family in the Saudi Arabian culture. More emphasis on the role of the family in end of life care decisions, including donation, should be considered as preface to the differing policy alternatives. This will enable the reader to truly understand the recommendations, why they are sound from a cultural-family perspective, and how the authors envision this to increase overall rates.”**

**Response:**

 I have added a preface that explains that the analysis sheds light on the cultural role of families in Saudi Arabia in the decision making process to provide the reader with context before discussing the alternatives.

 Comment: 

 “Opt-in

 The authors describe the current system which is a soft consent opt-in system; one in which a registered decision can be overturned by a family (Etheredge 2022). Updated statistics on the overall rate of registry would be recommended; the limited data on the success of ‘the App’ is impressive but stands on only 6 days of data and a very limited portion of the Saudi Arabian population. The overall argument against the status quo is not evident; exploration on the use/amount of public education would help justify the deviation from the status quo, for example.”

**Comment:**

**Thank you for your comment.**

**In terms of the app, which was newly launched at the time of writing this brief, more data on its usage has recently been published. We have included a recent study on the rates of organ donation registration versus the intention to highlight this gap, despite the app's launch, and to further justify the need to consider alternative consent systems.**

**Public education was also acknowledged as a potential way to aid in increasing the number of donors by focusing on the two areas that the study reported that the participants lacked knowledge on.  (Alhasan et al., 2023).**

**“Presumed consent system**

**Paragraph 2 requires literature to support/strengthen the last sentence on countries where this was not effective.**

**The last paragraph in this section becomes compelling on why this approach will not work in Saudi Arabia. To strengthen this point, I would suggest adding some literature on the impact to losing public trust if this method were to become policy. Additionally, data to support the last sentence is recommended.”**

**Response:**

 In terms of the second paragraph, I agree. I have omitted the last sentence since the evidence is sparse. I have put more emphasis on the ethical and cultural aspects as suggested to emphasize that this option is less likely favorable to the Saudi public.

**Comment:**

**“Mandated choice system**

**Capitalizing the Tawakkalna app requires data to demonstrate that the App is widely accessible for the large majority of the population.**

**Paragraph 4 is the beginning of a compelling argument for enhanced public awareness in combination with a consent system, not just mandated choice. The same could be stated for the status quo.**

**This section lacks cohesive flow. Paragraph 2 is incomplete; why does this approach ensure registration and information sharing with families? The last paragraph, sentence 2, begins to demonstrate why in Saudi Arabian culture, the use of mandated choice may be supported, “According to an Islamic belief, a good deed must be done with intent by the individual…”. Use this concept and literature that demonstrates how this improves autonomy of decisions to support the mandated choice approach, if this the best approach to be used.”**

**Comment:**

 -We thank you for pointing that out. To provide more clarification to the readers, information describing why the App is wildly used was added to the introduction section, including the number of registered donors. The App is used by the public for many essential services that the public use daily to access services from healthcare to civil affairs.  This clarification was added to the introduction. 

 -As for Paragraph 4 under informed consent, we have added an acknowledgement of the role of educational campaigns. 

 -Paragraph 2 was edited and a more recent study was added. In terms of information sharing with families, I have added a recommendation to add a feature on the App with an option to alert families about the donor’s wish.  

 -The last paragraph was rewritten to clarify how the religious belief in the necessity of conscious intent or consent to be a donor before death—so that it can be considered a good deed—could motivate individuals not only to register but also to reassure families that this was the conscious decision of their loved one as opposed to presumed consent where the donor may not have intended to donate.  This was an argument against presumed consent. Additionally, in previous paragraphs, I highlighted more evidence on why mandated choice could be preferred over informed consent which is why the recommendation was to explore mandated choice as an option. 

 -This section was also reorganized for a more cohesive flow as suggested. 

 Thank you.

# Reviewer report: Reviewer response for version 3

**DOI:** 10.5256/f1000research.149488.r202374

**Published:** 2023-09-20

**Authors:** Andreas Albertsen

**Affiliations:** 1 Aarhus University, Aarhus, Denmark

**Review of 'Changing the method of consent to increase the numbers of cadaveric donors in Saudi Arabia: the autonomy paradox'**

 This article interestingly presents the organ donation system in Saudi Arabia, and discuss various alternatives to the current model. In the end it recommends a policy change towards a mandated choice model. The arguments for this are partly normative (i.e. referring to weaknesses with alternative models, such as opt-in and opt-out), and partly of a more contextual nature, where it is argued that mandated choice is a better fit for Saudia Arabia than the alternatives (especially compared to opt-out). The authors write that mandated choice' could balance the respect for individual autonomy and the cultural role of families in Saudi Arabia'.

 I appreciate this kind of contribution and understand that there are clear limits to what can be done in the space of an article. Despite this, I think that there are still room for improvement both in terms of how the existing theoretical debate is presented and in terms of how the more context-based argument is made.

**Opt-in**

 In the discussion of opt-in (status quo) it would be worth mentioning that the register has only been in place for a few years, so we know little about its potential in the Saudi Arabian context – we do know, however, that it is often the case that many people don't register their preferences.

 It would improve this reader's understanding of the current system, if the family's role was described a bit more. Do they, for example, hold a so-called family veto? (That is, is the family allowed to over rule, even when people have registered their wish to donate?)

**Opt-Out**

 In the discussion of opt-out you could also cite Saunders' influential argument that people can provide tacit consent under such legislation (Saunders 2012). On such accounts, consent is not presumed.

 In terms of the effect of opt-out, most newer studies find that opt-out countries tend to do better than opt-in countries (Costa-Font, Rudisill, and Salcher-Konrad 2021; Steffel, Williams, and Tannenbaum 2019; Ugur and Molina Pérez 2023) – but much of this research is done on European data – so I would, at any rate – not want to use those findings to say something definitive about what to expect in Saudi Arabia (just like the authors do not want to do so).

 Author's write that opt-out 'might be unfeasible due to ethical considerations pertaining to the role of families' but this is unclear to me. Opt-out policies can be implemented in many different ways and can provide many different roles to the family (for a very useful overview, see (Molina-Pérez et al. 2022). Consider so-called soft opt-out legislation, where the family is allowed to decide if consent is presumed/tacit, but not when it is explicitly given. For example, the legislation in Wales (Albertsen 2018; Douglas and Cronin 2015). Note that many opt-out countries retain an organ donation register, and therefore, the possibility of opting-in. To make this argument convincing authors need to say more about the kind of reservations they have in mind among family members. For the argument to work, these reservations must hold across all specifications of an opt-out legislation, not just some.

 Another critique of opt-out is that it sends a signal about what the right thing to do is (Beraldo and Karpus 2021)

**Mandated Choice**

 Just to clarify: Would the proposed mandated choice policy only apply to this app's users? If so, what would happen with non-app users? Would there then just be an opt-in system for them?

 Authors write about the Australian example: 'the discontinuation of that policy was partially due to the public's opposition to this system's potential to increase the number of donors.' I don't quite understand what this means.

 What does it mean that people have not been given the opportunity to opt-out? Authors write: 'Furthermore, individuals are less likely to opt-out while alive if they have not been given the option of doing so, which may cause their families to consent after their deaths. 'And I don't see how the empirical claim in the following sentence is relevant for this. Please elaborate.

 I think the ethical concerns with mandated choice policies are addressed briefly. And please provide a Sunstein reference to the idea of a right not to choose (Sunstein 2014, 2015). Authors write, ' In terms of ethical concerns, opposing views of mandated choice argue that requiring people to make a choice could undermine autonomy by taking away the right not to choose.'

 The reply is that 'this system makes the two choices readily available; it increases a person's likelihood of making an autonomous choice and encourages self-determination.' But this does not change the fact that you are, forced to make a choice? Authors should recognize this. If you want to go deeper into the discussion about when such policies are autonomy-promoting, I have written an article about this (Thaysen and Albertsen 2021)

 In sum: This is an interesting and important article. More should be done to strengthen the arguments, and clarify certain elements both in terms of how the current system is portrayed and how the theoretical debate over alternatives is described.

**Minor:**

 I've learned that some find the term 'cadaveric donors' offensive and prefer deceased donors

Does the paper provide a comprehensive overview of the policy and the context of its implementation in a way which is accessible to a general reader?

Partly

Is the discussion on the implications clearly and accurately presented and does it cite the current literature?

Partly

Are the recommendations made clear, balanced, and justified on the basis of the presented arguments?

Yes

Reviewer Expertise:

Ethics of organ donation; political science, political philosophy

I confirm that I have read this submission and believe that I have an appropriate level of expertise to confirm that it is of an acceptable scientific standard, however I have significant reservations, as outlined above.

## References

[1] : Deemed consent: assessing the new opt-out approach to organ procurement in Wales. *J Med Ethics* .2018;44(5) : 10.1136/medethics-2017-104475 314-318 10.1136/medethics-2017-104475 29419421

[2] : Nudging to donate organs: do what you like or like what we do?. *Med Health Care Philos* .2021;24(3) : 10.1007/s11019-021-10007-6 329-340 10.1007/s11019-021-10007-6 33733389 PMC8349348

[3] : 'Relative Consent' or 'Presumed Consent'? Organ donation attitudes and behaviour. *Eur J Health Econ* .2021;22(1) : 10.1007/s10198-020-01214-8 5-16 10.1007/s10198-020-01214-8 32651683 PMC7822792

[4] : The Human Transplantation (Wales) Act 2013: an Act of Encouragement, not Enforcement. *The Modern Law Review* .2015;78(2) : 10.1111/1468-2230.12117 324-348 10.1111/1468-2230.12117

[5] : Should the family have a role in deceased organ donation decision-making? A systematic review of public knowledge and attitudes towards organ procurement policies in Europe. *Transplant Rev (Orlando)* .2022;36(1) : 10.1016/j.trre.2021.100673 100673 10.1016/j.trre.2021.100673 34864448

[6] : Opt-out organ donation without presumptions. *J Med Ethics* .2012;38(2) : 10.1136/medethics-2011-100039 69-72 10.1136/medethics-2011-100039 21828226

[7] : Does Changing Defaults save Lives? Effects of Presumed Consent Organ Donation Policies. *Behavioral Science & Policy* .2019;5(1) : 10.1177/237946151900500106 69-88 10.1177/237946151900500106

[8] : Choosing Not to Choose. *Duke Law Journal* .2014;1-52 25330554

[9] : Choosing Not to Choose: Understanding the Value of Choice. *New York, NY: Oxford University Press* .2015;

[10] : Mandated Choice Policies: When Are They Preferable?. *Political Research Quarterly* .2021;74(3) : 10.1177/1065912920936361 744-755 10.1177/1065912920936361

[11] : The toll of COVID-19 on organ donation and kidney transplantation in Europe: Do legislative defaults matter?. *Health Policy* .2023;136: 10.1016/j.healthpol.2023.104890 104890 10.1016/j.healthpol.2023.104890 37573724

# Response

**Published:** 2024-12-08

**Authors:** Deema AL Shawan

**Affiliations:** Imam Abdulrahman Bin faisal University, Saudi Arabia

Thank you for your constructive feedback on our policy brief. I have integrated your suggested edits into our revised manuscript, which enhanced the overall quality of the document. Below, you will find our point-by-point response.

 ____________________________

 Comment:

 “Opt-in

 In the discussion of opt-in (status quo) it would be worth mentioning that the register has only been in place for a few years, so we know little about its potential in the Saudi Arabian context – we do know, however, that it is often the case that many people don't register their preferences.”

 Response: 

 I completely agree, and I have clarified that the introduction of online registration is fairly recent.  However, in terms of how most individuals do not register their preference in terms of their willingness to be a donor, I did recommend mandated choice to ensure that users document their wishes via the Twakalna app. The majority of Saudis use this app to access many governmental services, therefore, it is a window of opportunity to implement our recommended policy. 

 Comment:

 “It would improve this reader's understanding of the current system, if the family's role was described a bit more. Do they, for example, hold a so-called family veto? (That is, is the family allowed to over rule, even when people have registered their wish to donate? “

 Response:

 That is correct. In Saudi Arabia, families have the right to veto the decision. I added a statement to clarify further for the reader.  Thank you for your comment.

 Comment:

 “In the discussion of opt-out you could also cite Saunders' influential argument that people can provide tacit consent under such legislation (Saunders 2012). On such accounts, consent is not presumed.

 In terms of the effect of opt-out, most newer studies find that opt-out countries tend to do better than opt-in countries (Costa-Font, Rudisill, and Salcher-Konrad 2021; Steffel, Williams, and Tannenbaum 2019; Ugur and Molina Pérez 2023) – but much of this research is done on European data – so I would, at any rate – not want to use those findings to say something definitive about what to expect in Saudi Arabia (just like the authors do not want to do so).

 Response:

 Thank you for your valuable insights and suggestions.

  Saunders’ argument about tacit consent in opt-out legislation provides an important perspective. As you mentioned, even though it was proved to be effective in opt-in countries in some countries in Europe, our argument situates this debate within the cultural context of Saudi Arabia.

 We also incorporated the other additions you suggested to strengthen our argument.

 Comment:

 “Author's write that opt-out 'might be unfeasible due to ethical considerations pertaining to the role of families' but this is unclear to me. Opt-out policies can be implemented in many different ways and can provide many different roles to the family (for a very useful overview, see (Molina-Pérez et al. 2022). Consider so-called soft opt-out legislation, where the family is allowed to decide if consent is presumed/tacit, but not when it is explicitly given. For example, the legislation in Wales (Albertsen 2018; Douglas and Cronin 2015). Note that many opt-out countries retain an organ donation register, and therefore, the possibility of opting-in. To make this argument convincing authors need to say more about the kind of reservations they have in mind among family members. For the argument to work, these reservations must hold across all specifications of an opt-out legislation, not just some.

 Another critique of opt-out is that it sends a signal about what the right thing to do is (Beraldo and Karpus 2021)”

 Response:

 We appreciate the nuanced arguments you presented; we incorporate them into our discussion.

 Comment:

 “Mandated Choice

 Just to clarify: Would the proposed mandated choice policy only apply to this app's users? If so, what would happen with non-app users? Would there then just be an opt-in system for them?”

 Response:

 Yes, the app is a national platform that is used by the majority of the population since it is used to access essential governmental services such as renewing national IDs and passports and even access to health records for the users and their dependents.  

 We also included this information and the number of users in the introduction to ensure that this information is clear to the reader.

 Comment: 

 “Authors write about the Australian example: 'the discontinuation of that policy was partially due to the public's opposition to this system's potential to increase the number of donors.' I don't quite understand what this means.”

 What does it mean that people have not been given the opportunity to opt-out? Authors write: 'Furthermore, individuals are less likely to opt-out while alive if they have not been given the option of doing so, which may cause their families to consent after their deaths. 'And I don't see how the empirical claim in the following sentence is relevant for this. Please elaborate.

 Response: 

 The statement was omitted and more arguments were added under the presumed consent alternative.  

 Comment:

 I think the ethical concerns with mandated choice policies are addressed briefly. And please provide a Sunstein reference to the idea of a right not to choose (Sunstein 2014, 2015). Authors write, ' In terms of ethical concerns, opposing views of mandated choice argue that requiring people to make a choice could undermine autonomy by taking away the right not to choose.'

 The reply is that 'this system makes the two choices readily available; it increases a person's likelihood of making an autonomous choice and encourages self-determination.' But this does not change the fact that you are, forced to make a choice? Authors should recognize this. If you want to go deeper into the discussion about when such policies are autonomy-promoting, I have written an article about this (Thaysen and Albertsen 2021)”

 Response:

 You brought up a significant point, I have added this concern to the mandated choice section of the brief as well as adjust the recommendation accordingly.

 Comment:

 “Minor:

 I've learned that some find the term 'cadaveric donors' offensive and prefer deceased donors”

 Response:

 Thank you for bringing this to our attention. We have corrected the term throughout the article as well as the title.

# Reviewer report: Reviewer response for version 2

**DOI:** 10.5256/f1000research.120509.r146557

**Published:** 2022-08-23

**Authors:** Alberto Molina-Pérez

**Affiliations:** 1 Institute for Advanced Social Studies, Spanish National Research Council, Cordoba, Spain

This is an improved version of the article. I appreciate the authors’ effort to address my concerns. However, several important issues remain. In particular, the justification for the recommendation to change the consent model to a mandatory choice system is confused from an argumentative point of view. Furthermore, the article does not provide sufficient evidence or good enough reasons to support the potential effectiveness of this change in achieving its objective.

**First, the recommendation of changing the consent system to address organ shortage is not sufficiently justified.**

 In the introduction, the article states that "family refusal is a major obstacle limiting donation" (33% of family consent in 2019) and that the "unknown wish of the donor is one of the reasons for the families' refusal to consent". Although this second claim is not based on studies of the Saudi population, it is very plausible. [By the way, a systematic review of bioethics in Saudi Arabia, including 33 studies on organ donation and transplantation, was published very recently and might contain relevant data to support this second claim (Woodman et al. 2022). I encourage the authors to consult this review if they have not already done so.] The article then states that "to overcome this issue, improvements should be made in documenting potential donors' consent in organ donation registries".

 So far, the rationale seems to be that since the shortage of transplantable organs is partly caused by high rates of family refusal, which is partly caused by unknown individual wishes, then improving the registration of individuals’ wishes may decrease family refusal rates and might, as a consequence, increase organ procurement. So far, so good.

 Then, the article introduces opt-in, opt-out and mandatory choice and states that "This paper aims to analyze the different types of consent systems to address the issue of the high rate of family refusal […]". There is an argumentative gap here. It is as if changing the consent model is the only possible solution to mitigate the problem of family refusals caused by unknown individual wishes. However, we know there are alternative policies that could be implemented. So, why are the authors focusing on this one alone? Is it supposed to be more effective, or easier to implement, or cheaper than alternative policies?

 In other words, among all the policies that could be implemented to try to increase individual registration and decrease family refusals, why is the article focusing only on changing the consent system?

**Second, the recommendation to move to a mandatory choice system is not adequately justified.**

 We will consider the three main reasons to move from opt-in to mandatory choice: effectiveness, ethics, and public support.

***Effectiveness***

 First, the article correctly mentions that the opt-in system is arguably not the most effective (in terms of converting willing donors into actual donors) because, in Saudi Arabia as in several other countries, many individuals do not document their willingness to donate.

 Then, the article suggests that the opt-out system is more effective by citing Rithalia and colleagues’ (2009) systematic review of the impact of opt-out. However, the article fail to mention that this study does not necessarily lead to this conclusion. According to Rithalia and colleagues: "The available evidence suggests that presumed consent is associated with increased organ donation rates, even when other factors are accounted for. However, it cannot be inferred from this that the introduction of presumed consent legislation per se will lead to an increase in organ donation rates." (Rithalia 2009, p.7)

 There are more recent studies and reviews on this topic. Some support the idea that moving from opt-in to opt-out contributes to increasing organ donation rates. Others don’t. At this time, this issue is not settled yet.

 Finally, the article mentions that implementing a mandated choice system and using the Tawakkalna app for this could increase the number of registered donors. The article also warns that "there is sparse evidence on the effectiveness of a mandated choice system in increasing the number of registered donors". Then, the article mentions a mandated choice policy in Australia that was discontinued because it was not successful.

 It is quite obvious that individuals who want to donate their organs are more likely to register their consent in a mandatory choice system than in an opt-in system, because they are required to register their wishes. As a consequence, families are more likely to know the deceased’s wishes. However, individuals who *do not* want to donate are also more likely to register their refusal in a mandatory choice system than in an opt-in system. Although registered consents will increase, registered refusals will increase too. Therefore, there is no guarantee that moving from opt-in to mandatory choice will lead to increased organ donation rates. 

 The recommendation of this article works on the paper, but it may not work in the real life. This article does not provide sufficient arguments or evidence to suggest that it might actually work. Arguably, changing legislation should not be a game of trial and error. As said in the abstract: "more research should be dedicated to investigating the Saudi public's current culture and perceptions of organ donation to ensure feasibility."

***Ethics***

 The article claims that a mandatory choice system might be the optimal solution in terms of balance between the respect for individual autonomy and the cultural role of the family in Saudi Arabia. So, why is it better than opt-in and opt-out?

 When examining the opt-in system from this perspective, the authors write the following:

 "Some argue that an opt-in system respects personal autonomy since this policy can ensure that the individual's wishes to become a donor is acknowledged (Farsides, 2012; Taylor, 2005). On the other hand, opponents of opt-in policies state that it could undermine autonomy by harvesting organs from unwilling donors who did not document their wishes. (Gill, 2004)."

 The second sentence is wrong: under an opt-in system those individuals who did not document their wishes are *not* considered as potential organ donors, because *by definition* in order to be considered as a potential donor, they must have opted in. By contrast, it is true that, under an *opt-out* system, organ can be procured from individuals who didn’t want to donate but failed to express their refusal.

 When examining the opt-out system, the authors write the following: 

 "A presumed consent system is ethically sound and may not violate autonomy; nevertheless, there are some concerns."

 There is a heated debate in the literature with regard to the ethical acceptability of presumed consent and whether or not it is respectful of individual autonomy. The very idea that someone’s consent could be presumed is problematic. Therefore, I wonder what reasons lead the authors to claim that presumed consent is ethically sound and may not violate autonomy.

 The article then mentions that "the presumption of consent is ’morally no worse than not removing organs from the bodies of people who did want them removed’ (Gill, 2004)". This sentence confuses two distinct issues. On the one hand, Michael Gill shows that there is presumption on both sides: opt-out presumes willingness to donate, opt-in presumes unwillingness to donate. There is no particular moral issue there. On the other hand, Gill argues that removing organs from the bodies of people who did not want them removed (opt-out) is "morally no worse than not removing organs from the bodies of people who did want them removed".

 Eventually, the article mentions the following: 

 "However, applying a presumed consent policy in the context of Saudi Arabia might be unfeasible due to ethical considerations pertaining to the role of families. In many cases, the family trumps an individuals' autonomy (Al-Shahri, 2002). Therefore, it may be culturally insensitive to implement a presumed consent system that disregards the wishes of the next of kin completely, which could risk public acceptance."

 There is a leap of argument between the second sentence and the third. The second sentence mentions the fact that the family can overrule the deceased’s wishes (trump an individual’s autonomy). This is actually quite common around the world and not specific to Saudi Arabia (Rosenblum et al. 2012; Delgado et al. 2019). The third sentence mentions that an opt-out system *that disregards the wishes of the next of kin completely* could risk public acceptance. Ok, but what about implementing an opt-out system that *does not* disregard the wishes of the next of kin? These soft opt-out systems are very common worldwide (Rosenblum et al. 2012; Delgado et al. 2019). If the family in Saudi Arabia is allowed to have the last word in the decision-making process under the opt-in system, the family may be allowed to have the last word under an opt-out system too. Besides, the authors’ remark would also apply to a mandated choice system *that disregards the wishes of the next of kin completely*.

 When examining the mandated choice system, the article mentions that "requiring people to make a choice could undermine autonomy by taking away the right not to choose" and that "nevertheless […], it increases a person's likelihood of making an autonomous choice and encourages self-determination". So, mandated choice encourages self-determination by taking away people’s right not to decide. Whether this is morally better or worse than the opt-in system is debatable.

***Public support***

 Finally, the article mentions that "a study conducted in Saudi Arabia ranked mandated choice as the most favored consent system", but warns that "the study was limited to a single tertiary hospital, and nation-wide studies must be conducted to further validate this finding".

**Summary**

 To summarise the rationale used in this article to recommend a mandated choice system: (1) It is unclear whether or not a mandated choice system would be more effective in increasing organ donation; (2) It is debatable whether a mandated choice system is morally better than opt-in; and (3) there is some but insufficient evidence to support that people in Saudi Arabia would prefer a mandated choice system better than opt-in, opt-out.

 This is not a compelling rationale.

 MINOR ISSUES

 "This result was partially due to the opposition to this system's potential to increase the number of donors who opt-out of being a donor."

 --> I don't understand this sentence.

 "One of the main obstacles to obtaining family consent in Saudi Arabia is family refusal."

 --> I don't know what this sentence is supposed to mean. That "family refusal is the main obstacle to family consent" is analytically true.

Does the paper provide a comprehensive overview of the policy and the context of its implementation in a way which is accessible to a general reader?

No

Is the discussion on the implications clearly and accurately presented and does it cite the current literature?

No

Are the recommendations made clear, balanced, and justified on the basis of the presented arguments?

Partly

Reviewer Expertise:

Ethics of organ donation; Health policy

I confirm that I have read this submission and believe that I have an appropriate level of expertise to state that I do not consider it to be of an acceptable scientific standard, for reasons outlined above.

## References

[1] : Current state of ethical challenges reported in Saudi Arabia: a systematic review & bibliometric analysis from 2010 to 2021. *BMC Med Ethics* .2022;23(1) : 10.1186/s12910-022-00816-6 82 10.1186/s12910-022-00816-6 35964019 PMC9375198

[2] : Impact of presumed consent for organ donation on donation rates: a systematic review. *BMJ* .2009;338: 10.1136/bmj.a3162 a3162 10.1136/bmj.a3162 19147479 PMC2628300

[3] : The authority of next-of-kin in explicit and presumed consent systems for deceased organ donation: an analysis of 54 nations. *Nephrol Dial Transplant* .2012;27(6) : 10.1093/ndt/gfr619 2533-46 10.1093/ndt/gfr619 22121233 PMC3363979

[4] : The Role of the Family in Deceased Organ Procurement: A Guide for Clinicians and Policymakers. *Transplantation* .103(5) : 10.1097/TP.0000000000002622 e112-e118 10.1097/TP.0000000000002622 31033855

# Response

**Published:** 2023-06-21

**Authors:** Deema AL Shawan

**Affiliations:** Imam Abdulrahman Bin faisal University, Saudi Arabia

Dear Peer Reviewer,

 Thank you for taking the time to review our policy brief. Once again, we appreciate your thoughtful feedback and are glad to hear that you found the brief well-written.

 We acknowledge that the evidence on the effectiveness of different registration consent methods is inconclusive. However, our brief aimed to frame the issue in a simplified manner and highlight implications for further research, specifically to collect more evidence on the effectiveness of mandated choice in Saudi Arabia.

 We would also like to clarify that our brief's aim was not to provide a comprehensive, in-depth ethical analysis but rather to provide a brief overview of the different considerations concerning Saudi culture.

 Nonetheless, we have incorporated some of your valuable suggestions into the revised version of the brief. We hope these revisions have addressed your concerns and improved the quality of our work.

 We appreciate your constructive criticism and feedback. 

 Best regards,

 Dr. Deema Saad Al Shawan

**The following is point-by-point response to your concerns:**

**-First, the recommendation of changing the consent system to address organ shortage is not sufficiently justified.**

**-Second, the recommendation to move to a mandatory choice system is not adequately justified.**

 Thank you for your input.

 We aimed to provide implications for further research since this topic is very understudied in Saudi Arabia.

**-Then, the article introduces opt-in, opt-out and mandatory choice and states that "This paper aims to analyze the different types of consent systems to address the issue of the high rate of family refusal […]". There is an argumentative gap here. It is as if changing the consent model is the only possible solution to mitigate the problem of family refusals caused by unknown individual wishes. However, we know there are alternative policies that could be implemented. So, why are the authors focusing on this one alone? Is it supposed to be more effective, or easier to implement, or cheaper than alternative policies?**

 We did not claim this was the only option. This paper highlights a window of opportunity that can occur by optimizing organ registration on the Twaklna App. The different efforts of the Saudi Center for Organ Donation are mentioned in the Background section of the memo, including awareness campiness.

**-There are more recent studies and reviews on this topic. Some support the idea that moving from opt-in to opt-out contributes to increasing organ donation rates. Others don’t. At this time, this issue is not settled yet.**

 We appreciate the feedback, and we have updated the manuscript to acknowledge that.

**-The second sentence is wrong: under an opt-in system those individuals who did not document their wishes are *not* considered as potential organ donors, because *by definition* in order to be considered as a potential donor, they must have opted in. By contrast, it is true that, under an *opt-out* system, organ can be procured from individuals who didn’t want to donate but failed to express their refusal.**

**Thank you for pointing this out. The phrasing of the second sentence was corrected.**

**Another article was cited with the correct clarification.**

 Thank you, we have made the correction as suggested. 

**MINOR ISSUES**

**-"This result was partially due to the opposition to this system's potential to increase the number of donors who opt-out of being a donor."**

**--> I don't understand this sentence.**

**-"One of the main obstacles to obtaining family consent in Saudi Arabia is family refusal."**

**--> I don't know what this sentence is supposed to mean. That "family refusal is the main obstacle to family consent" is analytically true.**

 Thank you, we have made the edits as suggested and clarified the manuscript.

# Reviewer report: Reviewer response for version 1

**DOI:** 10.5256/f1000research.79943.r136403

**Published:** 2022-06-20

**Authors:** James Stacey Taylor

**Affiliations:** 1 Department of Philosophy, The College of New Jersey, Ewing, NJ, USA

This is a nicely written paper that argues in favor of using mandated choice to increase the availability of transplant organs in Saudi Arabia. While it does not cover any new ground in its ethical discussion it provides helpful information about the ways in which new technology might be used to enhance organ procurement. However, it suffers from significant empirical and theoretical weaknesses that preclude its indexing as it stands, including inaccurate data, claims that are insufficiently supported by evidence and a poor grounding in philosophical ethics and action theory. I have provided an overview of these issues below.

**Issue 1:**

 “According to a news article, the number of registered donors increased from 20,000 to 200,000, which is a 115% increase after the launch of the donor registration feature.”

 Three points are worth noting here:

1. Is there a better source for these figures than a news article? Have the authors examined the original source on which this news article was based? How accurate is this claim?
2. The increase from 20,000 to 200,000 is not “115%” as the authors claim. This needs to be corrected.
3. When the authors write “…115% increase after the launch of the donor registration feature” do they mean that this 115% increase was immediate after the launch, and then numbers further increased, or do they mean to claim that the increase to 200,000 occurred in the total time after the launch? (The former construal would be compatible with there being an initial increase of 115% and then an greater increase subsequently.)

**Issue 2:**

 “As for ethical considerations, the current opt-in system respects an individual's autonomy. According to Immanuel Kant, undermining a person's autonomy is to treat that person as a mere means to an end without regard for the individual's own goals. Based on Kant's theory, organs from a deceased individual should not be harvested if the person's wish was not to be a donor, even if it intends to save others.”

 There are three points that should be made with respect to the claims in this sentence.

1. The authors should distinguish between personal autonomy and Kantian autonomy. On a theory of personal autonomy a person is autonomous with respect to her desires and actions if they flow from her motivational set in a way that ensures that they originate from her, rather than from elsewhere (where the requirements for what counts as originating from her are spelled out in the theory of personal autonomy that one adopts). On a Kantian account of autonomy a person is autonomous if (roughly) she acts out of respect for the moral law. These are distinct accounts of autonomy, and since the former is often of primary interest in discussions of organ procurement the authors would do well to focus on it rather than its Kantian alternative.
2. Why couldn’t an opt-out approach to organ procurement also respect a person’s (personal) autonomy? The authors should see here Michael Gill’s “Presumed Consent, Autonomy, and Organ procurement,” *Journal of Medicine and Philosophy* 29, 1 (2004): 37 – 59 ^1^.
3. Merely claiming that Kant would hold that a person’s organs should not be harvested after her death if she did not agree to this is insufficient. The authors need to argue that Kant (or Kantians) are committed to holding that persons have duties to respect the wishes of the dead.

**Issue 3:**

 The authors should note that the differences in the effectiveness of the Austrian and Spanish systems might also be a product of differing infrastructure that is in place to secure available organs, and not merely the result of their different approaches to opt-out systems. The authors also need to provide better sources for their claims here.

**Issue 4:**

 “According to a study conducted in Saudi Arabia in 2018, most participants support organ donation…” - What percentage is captured by “most”?

**Issue 5:**

 “As for ethical considerations, a mandated choice system makes the two choices readily available; it increases a person's likelihood of making an autonomous choice and encourages self-determination.”

 Why does requiring persons to make a choice between two alternatives increase her “…likelihood of making an autonomous choice and encourages self-determination”? Indeed, couldn’t requiring that a person choose between two alternatives decrease the degree to which she is autonomous with respect to her relevant choices, insofar as it removes from her the option not to choose — an option which some persons might have preferred? The authors need to do much more to support their claims here, especially since they appear prima facie implausible.

Does the paper provide a comprehensive overview of the policy and the context of its implementation in a way which is accessible to a general reader?

Partly

Is the discussion on the implications clearly and accurately presented and does it cite the current literature?

No

Are the recommendations made clear, balanced, and justified on the basis of the presented arguments?

Partly

Reviewer Expertise:

The ethics of organ procurement, autonomy theory, and the metaphysics of death.

I confirm that I have read this submission and believe that I have an appropriate level of expertise to state that I do not consider it to be of an acceptable scientific standard, for reasons outlined above.

## References

[1] : Presumed consent, autonomy, and organ donation. *J Med Philos* .2004;29(1) : 10.1076/jmep.29.1.37.30412 37-59 10.1076/jmep.29.1.37.30412 15449812

# Response

**Published:** 2022-07-05

**Authors:** Deema AL Shawan

**Affiliations:** Imam Abdulrahman Bin faisal University, Saudi Arabia

Dear Professor Taylor,

 Thank you for your insightful comments and suggestions. We have incorporated the edits to our manuscript. Additionally, we have a point-by-point response below.

**Comment: **“According to a news article, the number of registered donors increased from 20,000 to 200,000, which is a 115% increase after the launch of the donor registration feature.”

 “Is there a better source for these figures than a news article? Have the authors examined the original source on which this news article was based? How accurate is this claim?”

 When the authors write “…115% increase after the launch of the donor registration feature” do they mean that this 115% increase was immediate after the launch, and then numbers further increased, or do they mean to claim that the increase to 200,000 occurred in the total time after the launch? (The former construal would be compatible with there being an initial increase of 115% and then an greater increase subsequently.)

**Response:** This was a error on part of the news article itself, therefore, we have corrected the information and replaced it with a more reliable source.

**Comment: **“The authors should distinguish between personal autonomy and Kantian autonomy. On a theory of personal autonomy a person is autonomous with respect to her desires and actions if they flow from her motivational set in a way that ensures that they originate from her, rather than from elsewhere (where the requirements for what counts as originating from her are spelled out in the theory of personal autonomy that one adopts). On a Kantian account of autonomy a person is autonomous if (roughly) she acts out of respect for the moral law. These are distinct accounts of autonomy, and since the former is often of primary interest in discussions of organ procurement the authors would do well to focus on it rather than its Kantian alternative.

**Response:** We thank you for the insightful comment, we agree and we have made the suggested correction to focus on personal autonomy. We made the clarification in the background, analysis sections.

**Comment: **“Why couldn’t an opt-out approach to organ procurement also respect a person’s (personal) autonomy? The authors should see here Michael Gill’s “Presumed Consent, Autonomy, and Organ procurement,” Journal of Medicine and Philosophy 29, 1 (2004): 37 – 591.”

**Response:** Thank you for the insightful comment and sharing this reference. We have incorporated your suggestion into our manuscript and expressed the other potential concerns. For one thing, there is a risk that organs could be removed from bodies of individuals who did not want their organs removed. On the other hand, we do agree with (Gill, 2004) that “it is morally no worse than not removing organs from the bodies of people who did want them removed.”  However, as stated in the manuscript this method is not favored by the Saudi public due to religious reasons. (Hammami et al., 2012) Which is why we recommended that decision makers support investigating mandated choice as an option, since it could be a balance between the risk of removing organs from individuals who did not wish to be donors and the risk of not harvesting organs from willing donors. The clarification was made in the manuscripts and additional references were added.

**Comment:** “Merely claiming that Kant would hold that a person’s organs should not be harvested after her death if she did not agree to this is insufficient. The authors need to argue that Kant (or Kantians) are committed to holding that persons have duties to respect the wishes of the dead.”

**Response: **We have updated to individual autonomy as recommended in a previous comment and provided further explanations and evidence.

**Comment: **The authors should note that the differences in the effectiveness of the Austrian and Spanish systems might also be a product of differing infrastructure that is in place to secure available organs, and not merely the result of their different approaches to opt-out systems. The authors also need to provide better sources for their claims here.

**Response: **The sources were updated accordingly.

**Comment: **“According to a study conducted in Saudi Arabia in 2018, most participants support organ donation…” - What percentage is captured by “most”?

**Response:** The percentage was added as suggested.  

**Comment: **“Why does requiring persons to make a choice between two alternatives increase her “…likelihood of making an autonomous choice and encourages self-determination”? Indeed, couldn’t requiring that a person choose between two alternatives decrease the degree to which she is autonomous with respect to her relevant choices, insofar as it removes from her the option not to choose — an option which some persons might have preferred? The authors need to do much more to support their claims here, especially since they appear prima facie implausible.”

**Response: **That is a valid argument, however, we took into consideration that religion and culture play an important role in determining how autonomy is perceived. We made this recommendation considering the applicability to Saudi Arabia’s culture role of families, religion, public acceptance. Due to religious beliefs for organ donation to be a considered good deed there must be intent by the individual to be a donor. Due to this belief, clearly knowing the next of kin wish could influence the families to approve of the donation. Furthermore, according to a study we have added to the manuscript, mandated choice was the most favored option by the public. Lastly, due to the accessibility of the App, users have the option to opt-out at any moment.

 We look forward to hearing back from you and please let us know if you have any further inquiries and comments you may have.

 Sincerely,

 Dr. Deema S. Al Shawan

# Reviewer report: Reviewer response for version 1

**DOI:** 10.5256/f1000research.79943.r120048

**Published:** 2022-01-20

**Authors:** Alberto Molina-Pérez

**Affiliations:** 1 Institute for Advanced Social Studies, Spanish National Research Council, Cordoba, Spain

The article first describes the organ donation system in Saudi Arabia and identifies the high rate of family opposition as one major obstacle to increasing cadaveric organ donation rates in the country. Then, the article presents the three main consent policies: opt-in (explicit consent), opt-out (presumed consent), and mandated choice, advocating for the latter one as the best alternative in the context of Saudi Arabia.

 Overall, the article is well written and provides a clear and accessible overview of the consent policy alternatives. One interesting point mentioned in the article is the use of the Tawakkalna app as a means of registration of individual preferences about organ donation. However, the article lacks depth in its analysis and suffers from some mistakes and inaccuracies that need to be addressed.

***"Does the paper provide a comprehensive overview of the policy and the context of its implementation in a way which is accessible to a general reader?"***

 Only partly. The overview is accessible to a general reader but it is not comprehensive and it lacks depth in its analysis. Also, its main hypothesis is based on insufficient evidence.

 For instance, after mentioning that only 33% of approached families authorised in 2019 the retrieval of organs from their loved ones, the article claims that the relatives’ lack of knowledge of the deceased’s wishes as one of the main reasons for the families’ refusal. [By the way, the wording of the sentence beginning "Lack of knowledge of the potential donor…" should be revised.] This second claim is based on Palmer 2012. Although this reference appears to be a good review of the literature, it has not been peer-reviewed. In addition, it is also relatively outdated (meaning that other reviews have been conducted more recently) and the studies reviewed were conducted mostly in English speaking Western countries: the United States, the United Kingdom, Australia, which means that their conclusions may not necessarily be extensible to other cultural contexts such as Saudi Arabia. In other words, although it may be true that lack of knowledge by the family is, in these countries, one of the main reasons for their refusal, there is no guarantee that the same conclusion holds in other countries, especially when these countries (e.g. Saudi Arabia) have a different social organization, religion, and cultural background.

 Since the argument of the article is based on the claim that family refusals are mainly caused by the family’s lack of knowledge of the potential donor wishes (which could be addressed, according to the authors, by a mandated choice policy), stronger evidence is needed to support this claim, especially in the context of Saudi Arabia. In other words, authors should provide more updated and compelling evidence that this claim is true—or, at the very least, that it *may* be true in their country—before proposing a policy to address it. 

 There is a rich and vast literature of empirical studies on the factors of family decision-making and willingness/refusal to donate that are contemporary or posterior to Palmer 2012. Here are some peer-reviewed literature reviews I am aware of, including some systematic reviews:

- Kentish-Barnes N, Siminoff LA, Walker W, Urbanski M, Charpentier J, Thuong M, et al. A narrative review of family members’ experience of organ donation request after brain death in the critical care setting. *Intensive Care Med* 2019;45:331–42. ^1^
- Miller C, Breakwell R. What factors influence a family’s decision to agree to organ donation? A critical literature review. *London Journal of Primary Care* 2018;10:103–7. ^2^
- Makmor T, Abdillah N, NurulHuda Ms, Raja Noriza RA, Roza Hazli Z. Family Decision-Making About Organ Donation: A Systematic Review. *JUMMEC* 2015;18:1–4. ^3^
- Ralph A, Chapman JR, Gillis J, Craig JC, Butow P, Howard K, et al. Family Perspectives on Deceased Organ Donation: Thematic Synthesis of Qualitative Studies. *American Journal of Transplantation* 2014;14:923–35. ^4^
- Walker W, Broderick A, Sque M. Factors Influencing Bereaved Families’ Decisions About Organ Donation: An Integrative Literature Review. *West J Nurs Res* 2013;35:1339–59. ^5^.
- de Groot J, Vernooij-Dassen M, Hoedemaekers C, Hoitsma A, Smeets W, van Leeuwen E. Decision making by relatives about brain death organ donation: an integrative review. *Transplantation* 2012;93:1196–211. ^6^
- Irving MJ, Tong A, Jan S, Cass A, Rose J, Chadban S, et al. Factors that influence the decision to be an organ donor: a systematic review of the qualitative literature. *Nephrology Dialysis Transplantation* 2012;27:2526–33. ^7^

Other relevant reviews and recent articles include:

- Curtis RMK, Manara AR, Madden S, Brown C, Duncalf S, Harvey D, et al. Validation of the factors influencing family consent for organ donation in the UK. *Anaesthesia* 2021:anae.15485. ^8^
- López JS, Soria-Oliver M, Aramayona B, García-Sánchez R, Martínez JM, Martín MJ. An Integrated Psychosocial Model of Relatives’ Decision About Deceased Organ Donation (IMROD): Joining Pieces of the Puzzle. Front Psychol 2018;9. ^9^
- López JS, Martínez JM, Soria-Oliver M, Aramayona B, García-Sánchez R, Martín MJ, et al. Bereaved relatives’ decision about deceased organ donation: An integrated psycho-social study conducted in Spain. Social Science & Medicine 2018;205:37–47. ^10^
- Chandler JA, Connors M, Holland G, Shemie SD. “Effective” Requesting: A Scoping Review of the Literature on Asking Families to Consent to Organ and Tissue Donation. *Transplantation* 2017;101:S1–16. ^11^
- Shah SK, Kasper K, Miller FG. A narrative review of the empirical evidence on public attitudes on brain death and vital organ transplantation: the need for better data to inform policy. Journal of Medical Ethics; London 2015;41. ^12^
- Sharp C, Randhawa G. Altruism, gift giving and reciprocity in organ donation: A review of cultural perspectives and challenges of the concepts. *Transplantation Reviews* 2014;28:163–8. ^13^
- Siminoff LA, Agyemang AA, Traino HM. Consent to organ donation: a review. *Progress in Transplantation* 2013;23:99–104. ^14^

After mentioning the lack of evidence as "one of the main reasons for the families’ refusal to consent", the authors write: "To overcome this issue, improvements should be made in documenting potential donors’ consent in organ donation registries." This conclusion does not necessarily follow or only partially. There are several other means to address family refusal rates, on the one hand, and the lack of knowledge issue, on the other. These means include mass media campaigns, targeted educational campaigns, the presence of a Specialist Nurse for Organ Donation (SNOD) as it is done in the UK, extensive training of transplant coordinators for the family interview as it is done in Spain, financial incentives to donation, prioritization programmes as it is done in Israel, etc. Some countries (e.g. Germany) with much lower family refusal rates than Saudi Arabia, do not have an organ donation registry but only an organ donor card. Other countries (e.g. Spain) with very low family refusal rates, do not have any registry at all (except for living wills that are barely used in practice for registering organ donation preferences) nor any official organ donor card. Fostering communication about organ donation among relatives and training the medical staff to conduct the family interview are alternative ways to increase family knowledge without requiring the registration or documentation of individual preferences.

 In the next paragraph, the article says: "There are three known methods of consent when registering to become a donor upon death: informed consent (opt-in system), mandated choice and presumed consent (opt-out system)." This wording is confusing. First, the opt-in system is not based on *informed* consent but only on *explicit* or *expressed* consent. Second, although the opt-out system is compatible with a registry of refusals, presumed consent only applies when *no registering* has taken place. Third, it is important to differentiate the models of individual consent (informed, presumed) and consent policies (opt-in, opt-out, mandated choice) from the means available to individuals to express their consent or refusal to donate. Some countries have registries, others don’t, regardless of their consent policy.

 The article then states its main objective: "This paper aims to analyze the different types of consent systems to address the issue of the high rate of family refusal. The study offers some important insights that will aid decision-makers in selecting an alternative that is more likely to reduce the shortage of organs."

 This objective presupposes that there is a causal relationship between the consent systems and the rates of family refusal. I would recommend the authors provide references or data to support such an assumption. I ignore whether this is the case or not and whether there is at least a correlation between the two. I am really curious about it. By looking at the Global Observatory on Donation and Transplantation’s Newsletter Transplant 2017, which provides data on family refusal in many countries throughout the world, I fail *prima facie* to see any trend that may be related to the consent systems in place.

 With regard to the article’s proposal of a mandated choice policy, it is true that if registering a preference in favour or against organ donation was mandatory in the country, this would likely increase the registration rates and decrease the families’ lack of knowledge about their loved one’s wishes. As a consequence, the number of family refusals caused by this lack of knowledge would also likely decrease. Question is: first, is this the best way to increase organ donation rates and, second, how many family refusals are actually caused by lack of knowledge in Saudi Arabia?

 To answer these questions, it would be good to give some perspective. According to the Global Observatory on Donation and Transplantation, Saudi Arabia had a deceased organ donation rate of 3.3 pmp in 2019 and 1.9 pmp in 2020, which is relatively low although not in the lower end of the ranking. In 2017, according to the same source, Saudi Arabia had a population of 32.2 M and a total of 92 deceased donors, that is, a rate of 2.9 donors pmp; 333 family interviews were conducted asking for authorisation, out of which 230 (69%) refused. The article mentions that, according to a 2009 study of Saudi University students, "about 70% of participants are willing to become donors". If this figure was representative of the general population, if all individuals had registered their wishes, if all families of those willing to donate authorised organ procurement, and if all potential donors were eventually converted into actual donors, the total number of donors in 2017 would have been 92 + (333 x 70%) = 325, increasing the donors rate to 10% which is much better (comparable to that of Germany that same year). [Another study (Alnasyan et al. 2019) also cited in the article but whose figures on willingness to donate are not mentioned by the article says that 77.7% of the general public expressed a willingness to donate, although 29.1% were willing to donate only to their relatives]

 The problem is that we don’t know how likely is such a scenario. There are no data provided in the article regarding how many of the family refusals were caused by lack of knowledge or by other reasons such as those cited in Palmer 2012: Not wishing for surgery to the body/having concerns regarding disfigurement; Feelings that the patient had suffered enough; Disagreements among the family group; Religious/cultural reasons; Dissatisfaction with healthcare staff and process; Concerns over delay to funeral/burial process; Unable to accept death, or lack of understanding of brain death; Concerns regarding the integrity of process e.g. unfair organ allocation of organs and organ selling; Relatives deciding themselves that organs would not be suitable; Longstanding negative views on organ donation; Relatives were emotionally exhausted.

 Actually, the article itself mentions that some unknown proportion of the family refusals may not be caused by lack of knowledge: "It is important to note that, under the current policy, families still have the right to veto that decision despite their loved one’s consent to become a donor." Also: "In many cases, the family trumps an individual’s autonomy (Al-Shahri, 2002)" The reference here cited (Al-Shahri, 2002) says "In Saudi culture, the authority of the family overrules the individuals’ autonomy. Decisions taken by patients can often be altered according to the views of the family". I am thus curious to know the proportion of families who veto the deceased’s consent as well as their reasons to do so, because this is not caused by lack of knowledge of the deceased’s wishes, and some of the families who do lack knowledge may share the same reasons to refuse as those who don’t.

***Is the discussion on the implications clearly and accurately presented and does it cite the current literature?***

 The discussion on the implications is quite clear but it lacks accuracy and it does not cite enough updated and relevant literature.

 For example, the authors mention that "According to a news article, the number of registered donors increased from 20,000 to 200,000, which is a 115% increase after the launch of the donor registration feature". This is a ten-fold increase and definitely not a 115% increase. Something is wrong. I checked (using an automated translator) the news article cited here and it seems that these figures are actually mentioned. I failed to see any sources cited in the news article. This tells me that this news article is not reliable and thus it is not worth citing in a scientific article. Anyway, even if the 20,000 to 200,000 increase in registrations was true, compared to more than 34 M inhabitants in 2021, this represents only 0.6% of the total population. This is much better, but still anecdotal.

 Another example. The article claims that "after adopting a hard opt-out policy in Austria, the rate of donations quadrupled within eight years (Zink *et al*., 2005). On the other hand, a soft approach to the policy did not impact donation rates in Spain, which indicates that a hard opt-out policy could be more effective." There are several problems here. First, the reference cited with regard to Austria does not provide any evidence for the claim but cites two other sources, one of which ^15^ makes this claim without any evidence or references provided. This claim may be true or it may not, but the reference cited here is not sufficient to support the claim. Second, with regard to Spain, the policy is soft in clinical practice but hard according to the law. Third, what did impact donation rates in Spain was not the implementation of an opt-out policy in 1979, but the creation of the National Transplant Organisation (ONT) in 1989. Fourth, it is odd to claim that a hard opt-out policy (such as in Austria) could be more effective than a soft opt-out policy (such as in Spain) considering that Spain has a much higher donation rate (49.6 pmp in 2019) than Austria (23.4 pmp in 2019) according to the GODT. Fifth, several studies, including systematic reviews, have explored the impact of presumed consent policies. They should be cited.

 One more example. The article mentions "a study in the Netherlands, a country that adopted a mandated choice, concluded that this system generated more registered donors than both informed and presumed consent systems (Van Dalen & Henkens, 2014)." First, the Netherlands had *not* adopted a mandated choice policy but an opt-in system (as the authors of the cited study clearly state in their article). Second, the Netherlands have recently implemented an opt-out system. Third, the cited study does not assess the actual performance of a mandated choice system in the country but compares, in an experimental setting using counterfactuals, whether people would register as donors (hypothetically) if they moved to a region with presumed consent, explicit consent, or mandated choice. Fourth, the results from this study show that people would be more willing to register as donors under a mandated choice system (66%), but this result is only 4 points higher than presumed consent (62%) and many more people would also refuse to donate under a mandated choice system (34%) than under a presumed consent system (18%). In other words, mandated choice would increase donors by 4% and non-donors by 16% when compared to presumed consent.

***Are the recommendations made clear, balanced, and justified on the basis of the presented arguments?***

 The first recommendation is changing the method of obtaining consent from opt-in to mandated choice. Considering the comments above, it is not sufficiently justified. The authors may want to look at a similar recommendation made in other countries, such as a law proposal (unsuccessful) made by members of the German Parliament (see http://dip21.bundestag.de/dip21/btd/19/110/1911087.pdf, text in German), as well as the report on organ donation by the Swiss National Human Medical Ethics Commission (see https://www.nek-cne.admin.ch/inhalte/Themen/Stellungnahmen/fr/NEK-Stellungnahme_Organspende_FR.pdf, text in French).

 The second recommendation refers to modifying the Tawakkalna App. The proposed modifications may be useful, provided that more people register their organ donation preferences in the app. 

 The third recommendation or part of it is sensible: "more research dedicated to assessing the feasibility of implementing a new consent system", as well as "understanding the current Saudi context" to predict [I would say, estimate] its success.

Does the paper provide a comprehensive overview of the policy and the context of its implementation in a way which is accessible to a general reader?

No

Is the discussion on the implications clearly and accurately presented and does it cite the current literature?

No

Are the recommendations made clear, balanced, and justified on the basis of the presented arguments?

Partly

Reviewer Expertise:

Ethics of organ donation; Health policy

I confirm that I have read this submission and believe that I have an appropriate level of expertise to state that I do not consider it to be of an acceptable scientific standard, for reasons outlined above.

## References

[1] : A narrative review of family members' experience of organ donation request after brain death in the critical care setting. *Intensive Care Med* .45(3) : 10.1007/s00134-019-05575-4 331-342 10.1007/s00134-019-05575-4 30840119

[2] : What factors influence a family's decision to agree to organ donation? A critical literature review. *London J Prim Care (Abingdon)* .2018;10(4) : 10.1080/17571472.2018.1459226 103-107 10.1080/17571472.2018.1459226 30083243 PMC6074665

[3] : FAMILY DECISION-MAKING ABOUT ORGAN DONATION: A SYSTEMATIC REVIEW. *Journal of Health and Translational Medicine* .2015;18(1) : 10.22452/jummec.vol18no1.1 1-4 10.22452/jummec.vol18no1.1

[4] : Family perspectives on deceased organ donation: thematic synthesis of qualitative studies. *Am J Transplant* .2014;14(4) : 10.1111/ajt.12660 923-35 10.1111/ajt.12660 24612855

[5] : Factors influencing bereaved families' decisions about organ donation: an integrative literature review. *West J Nurs Res* .2013;35(10) : 10.1177/0193945913484987 1339-59 10.1177/0193945913484987 23618820

[6] : Decision making by relatives about brain death organ donation: an integrative review. *Transplantation* .2012;93(12) : 10.1097/TP.0b013e318256a45f 1196-211 10.1097/TP.0b013e318256a45f 23318303

[7] : Factors that influence the decision to be an organ donor: a systematic review of the qualitative literature. *Nephrol Dial Transplant* .2012;27(6) : 10.1093/ndt/gfr683 2526-33 10.1093/ndt/gfr683 22193049

[8] : Validation of the factors influencing family consent for organ donation in the UK. *Anaesthesia* .76(12) : 10.1111/anae.15485 1625-1634 10.1111/anae.15485 33860929

[9] : An Integrated Psychosocial Model of Relatives' Decision About Deceased Organ Donation (IMROD): Joining Pieces of the Puzzle. *Frontiers in Psychology* .2018;9: 10.3389/fpsyg.2018.00408 10.3389/fpsyg.2018.00408 29692744 PMC5902731

[10] : Bereaved relatives' decision about deceased organ donation: An integrated psycho-social study conducted in Spain. *Soc Sci Med* .205: 10.1016/j.socscimed.2018.03.039 37-47 10.1016/j.socscimed.2018.03.039 29631200

[11] : "Effective" Requesting: A Scoping Review of the Literature on Asking Families to Consent to Organ and Tissue Donation. *Transplantation* .101(5S Suppl 1) : 10.1097/TP.0000000000001695 S1-S16 10.1097/TP.0000000000001695 28437367

[12] : A narrative review of the empirical evidence on public attitudes on brain death and vital organ transplantation: the need for better data to inform policy. *J Med Ethics* .2015;41(4) : 10.1136/medethics-2013-101930 291-6 10.1136/medethics-2013-101930 24769621

[13] : Altruism, gift giving and reciprocity in organ donation: a review of cultural perspectives and challenges of the concepts. *Transplant Rev (Orlando)* .2014;28(4) : 10.1016/j.trre.2014.05.001 163-8 10.1016/j.trre.2014.05.001 24973193

[14] : Consent to organ donation: a review. *Prog Transplant* .2013;23(1) : 10.7182/pit2013801 99-104 10.7182/pit2013801 23448829 PMC6776471

[15] : Presumed consent: an international comparison and possibilities for change in the United States. *Camb Q Healthc Ethics* .2005;14(1) : 10.1017/s0963180105050139 113-8 10.1017/s0963180105050139 15747489

# Response

**Published:** 2022-07-05

**Authors:** Deema AL Shawan

**Affiliations:** Imam Abdulrahman Bin faisal University, Saudi Arabia

Dear Dr. Molina-Pérez,

 We thank you for taking the time to provide constructive feedback on our policy brief. We have incorporated the suggested edits to reflect your valuable input into our resubmitted manuscript. Additionally, we have a point-by-point response below:

**Comment: **“This second claim is based on Palmer 2012. Although this reference appears to be a good review of the literature, it has not been peer-reviewed. In addition, it is also relatively outdated (meaning that other reviews have been conducted more recently) and the studies reviewed were conducted mostly in English speaking Western countries: the United States, the United Kingdom, Australia, which means that their conclusions may not necessarily be extensible to other cultural contexts such as Saudi Arabia.”

**Response:** We agree, and to support the claim, we have replaced the (Palmer, 2012) article with one of your suggested sources

**Comment: **“In other words, although it may be true that lack of knowledge by the family is, in these countries, one of the main reasons for their refusal, there is no guarantee that the same conclusion holds in other countries, especially when these”

 “Since the argument of the article is based on the claim that family refusals are mainly caused by the family’s lack of knowledge of the potential donor wishes (which could be addressed, according to the authors, by a mandated choice policy), stronger evidence is needed to support this claim, especially in the context of Saudi Arabia. In other words, authors should provide more updated and compelling evidence that this claim is true—or, at the very least, that it *may* be true in their country—before proposing a policy to address it. “

**Response: **Due to the lack of up-to-date evidence in Saudi Arabia we have added evidence from another country as well as a survey about the Saudi publics acceptance towards this policy. Additionally, we have edited the recommendation to investigating it as a potential viable and feasible solution given the country’s culture.

**Comment: **“There are several other means to address family refusal rates, on the one hand, and the lack of knowledge issue, on the other. These means include mass media campaigns, targeted educational campaigns, the presence of a Specialist Nurse for Organ Donation (SNOD) as it is done in the UK, extensive training of transplant coordinators for the family interview as it is done in Spain, financial incentives to donation, prioritization programmes”

**Response: **We have acknowledged that there is a need to combine the proposed consent system with other methods to ensure it’s effectiveness to increase its effectiveness in reducing the shortage of organs

**Comment: **“In the next paragraph, the article says: "There are three known methods of consent when registering to become a donor upon death: informed consent (opt-in system), mandated choice and presumed consent (opt-out system)." This wording is confusing. “

**Response: **Thank you for pointing that out, we have corrected the wording accordingly.

**Comment:** “The article then states its main objective: "This paper aims to analyze the different types of consent systems to address the issue of the high rate of family refusal. The study offers some important insights that will aid decision-makers in selecting an alternative that is more likely to reduce the shortage of organs.This objective presupposes that there is a causal relationship between the consent systems and the rates of family refusal. I would recommend the authors provide references or data to support such an assumption. I ignore whether this is the case or not and whether there is at least a correlation between the two. I am really curious about it.”

**Response:** We appreciate the suggestion, even though we have not included a study that discusses the overall impact of consent systems on organ donation rates. We have included evidence on the effectiveness of each of the types of consent mechanisms throughout the policy brief.

**Comment:** “Question is: first, is this the best way to increase organ donation rates and, second, how many family refusals are actually caused by lack of knowledge in Saudi Arabia?”

**Response: **Thank you for your comment to clarify, the article does not claim that this is the best or the only approach but it is one of the main factors identified and it is also one of the most understudied. Additionally, as stated previously, we recommended  the incorporation of other interventions, such as educational campaigns, since changing the method of consent alone may not be as effective (justification and evidence were included in the manuscript). As for the second question, there are no up to date published statistics on the exact number of family refusal due to the lack of knowledge. Nevertheless, we have included a study by (Kentish-Barnes et al., 2019; Walker et al., 2013) that states that one of the causes of family refusal is not knowing their loved ones’ wish to donate.

**Comment:** *“*For example, the authors mention that "According to a news article, the number of registered donors increased from 20,000 to 200,000, which is a 115% increase after the launch of the donor registration feature". This is a ten-fold increase and definitely not a 115% increase. Something is wrong. I checked (using an automated translator) the news article cited here and it seems that these figures are actually mentioned. I failed to see any sources cited in the news article. This tells me that this news article is not reliable and thus it is not worth citing in a scientific article. Anyway, even if the 20,000 to 200,000 increase in registrations was true, compared to more than 34 M inhabitants in 2021, this represents only 0.6% of the total population. This is much better, but still anecdotal.

**Response: **The article was replaced with on of the few reliable published sources. The information was also edited to demonstrate the rapid registrations after the launch of the app instead of the claim that the app has increased the number of donors due to the lack of evidence. This was to further support the objective of the paper which is to urge decision-makers to take the opportunity to optimize the newly launched feature in the app in the future

**Comment: **“the reference cited with regard to Austria does not provide any evidence for the claim but cites two other sources, one of which ^15^ makes this claim without any evidence or references provided. This claim may be true or it may not, but the reference cited here is not sufficient to support the claim. “

 “Fourth, it is odd to claim that a hard opt-out policy (such as in Austria) could be more effective than a soft opt-out policy (such as in Spain) considering that Spain has a much higher donation rate (49.6 pmp in 2019) than Austria (23.4 pmp in 2019) according to the GODT.

 “several studies, including systematic reviews, have explored the impact of presumed consent policies. They should be cited.”

**Response:** As recommended, the study was replaced by by a systematic review that investigated the impact of the presumed consent policy in different countries. The other other findings were also corrected as suggested.

**Comment:** “the Netherlands had *not* adopted a mandated choice policy but an opt-in system “

 “Third, the cited study does not assess the actual performance of a mandated choice system in the country but compares, in an experimental setting using counterfactuals, whether people would register as donors (hypothetically) if they moved to a region with presumed consent, explicit consent, or mandated choice. “

 “the results from this study show that people would be more willing to register as donors under a mandated choice system (66%), but this result is only 4 points higher than presumed consent (62%) and many more people would also refuse to donate under a mandated choice system (34%) than under a presumed consent system (18%). In other words, mandated choice would increase donors by 4% and non-donors by 16% when compared to presumed consent.

**Response: **We thank you for the corrections and suggestions, the sources were updated.

 We look forward to hearing from you regarding our submission and to respond to any further inquiries and comments you may have.

 Sincerely,

 Dr. Deema S. Al Shawan
